# Environmental factors have a greater influence on photosynthetic capacity in C_4_ plants than biochemical subtypes or growth forms

**DOI:** 10.1111/nph.70525

**Published:** 2025-08-31

**Authors:** Yuzhen Fan, Daniel W. A. Noble, Belinda E. Medlyn, Russell K. Monson, Rowan F. Sage, Nicholas G. Smith, Elizabeth A. Ainsworth, Florian A. Busch, Florence R. Danila, Maria Ermakova, Patrick Friesen, Robert T. Furbank, Shu Han Gan, Oula Ghannoum, Daniel M. Griffith, Lianhong Gu, Vinod Jacob, Jürgen Knauer, Andrew D. B. Leakey, Shuai Li, Danica L. Lombardozzi, Martha Ludwig, Varsha S. Pathare, Murilo M. Peixoto, Karine Prado, Balasaheb V. Sonawane, Christopher J. Still, Susanne von Caemmerer, Russell Woodford, Danielle A. Way

**Affiliations:** ^1^ Division of Plant Sciences, Research School of Biology The Australian National University Canberra ACT 2601 Australia; ^2^ Division of Ecology and Evolution, Research School of Biology The Australian National University Canberra ACT 2601 Australia; ^3^ Hawkesbury Institute for the Environment Western Sydney University Penrith NSW 2751 Australia; ^4^ Department of Ecology and Evolutionary Biology University of Colorado Boulder CO 80309 USA; ^5^ Department of Ecology and Evolutionary Biology University of Toronto Toronto ON M5R3C5 Canada; ^6^ Department of Biological Sciences Texas Tech University Lubbock TX 79409 USA; ^7^ Center for Advanced Bioenergy and Bioproducts Innovation University of Illinois at Urbana‐Champaign Urbana IL 61801 USA; ^8^ Carl R. Woese Institute for Genomic Biology University of Illinois at Urbana‐Champaign Urbana IL 61801 USA; ^9^ Department of Plant Biology and Crop Sciences University of Illinois Urbana‐Champaign Urbana IL 61801 USA; ^10^ School of Biosciences and Birmingham Institute of Forest Research University of Birmingham Birmingham B15 2TT UK; ^11^ School of Biological Sciences Monash University Melbourne VIC 3800 Australia; ^12^ BioChambers Winnipeg MB R2W 3A8 Canada; ^13^ Department of Ecology, Evolution & Environmental Biology Columbia University New York NY 10027 USA; ^14^ Department of Ecology and Evolution Stony Brook University Stony Brook NY 11794 USA; ^15^ Environmental Sciences Division and Climate Change Science Institute Oak Ridge National Laboratory Oak Ridge TN 37831 USA; ^16^ School of Life Sciences, Faculty of Science University of Technology Sydney Ultimo NSW 2007 Australia; ^17^ Guangdong Provincial Key Laboratory of Applied Botany, South China Botanical Garden Chinese Academy of Sciences Guangzhou Guangdong 510650 China; ^18^ Department of Ecosystem Science & Sustainability Colorado State University Fort Collins CO 80523 USA; ^19^ Climate and Global Dynamics Laboratory National Center for Atmospheric Research Boulder CO 80305 USA; ^20^ School of Molecular Sciences University of Western Australia Perth WA 6009 Australia; ^21^ Soil and Crop Sciences Section Cornell University Ithaca NY 14853 USA; ^22^ School of Biological Sciences Washington State University Pullman WA 99164 USA; ^23^ Programa de Pós‐graduação em Biodiversidade Vegetal Universidade Federal de Goiás Instituto de Ciências Biológicas Goiânia GO 74690‐900 Brazil; ^24^ Plant Resilience Institute Michigan State University East Lansing MI 48824 USA; ^25^ Department of Biochemistry and Molecular Biology Michigan State University East Lansing MI 48824 USA; ^26^ Department of Plant Biology Michigan State University East Lansing MI 48824 USA; ^27^ Department of Plant, Soil, and Microbial Sciences Michigan State University East Lansing MI 48824 USA; ^28^ Department of Forest Ecosystems and Society Oregon State University Corvallis OR 97331 USA; ^29^ Department of Biology The University of Western Ontario London ON N6A3K7 Canada; ^30^ Nicholas School of the Environment Duke University Durham NC 27708 USA

**Keywords:** *A*/*C*_i_ curve, *A*_max_, C_4_ biochemical subtype, C_4_ photosynthesis, environmental response, photosynthesis modelling, *V*_pmax_

## Abstract

- Our understanding of how photosynthetic capacity varies among C_4_ species and across growth and measurement conditions remains limited.
- We collated 1696 CO_2_ response curves of net CO_2_ assimilation rate (*A*/*C*_i_ curves) from C_4_ species grown and measured at various environmental conditions and used these data to estimate the apparent maximum carboxylation activity of phosphoenolpyruvate carboxylase (*V*_pmaxA_) and CO_2_‐saturated net photosynthetic rate (*A*_max_), two key parameters describing photosynthetic capacity. We examined how *V*_pmaxA_ and *A*_max_ vary with species‐specific traits, growth and measurement conditions.
- We found little systematic variation of *V*_pmaxA_ and *A*_max_ across the classical C_4_ biochemical subtypes or growth forms, but showed that growth temperature and measurement conditions are major factors determining C_4_ photosynthetic capacity. We found no evidence that common C_4_ model species (e.g. maize, sorghum and *Setaria viridis*) differ in photosynthetic capacity from other C_4_ species when grown in controlled environments. However, C_4_ model species showed up to twice the photosynthetic capacity of other C_4_ species when grown in the field.
- Our multivariate model accounts for 47–51% of the variation reported in *V*_pmaxA_ and *A*_max_, and we argue that environmental conditions have a greater influence on C_4_ photosynthetic capacity than biochemical subtypes or growth forms.

## Introduction

C_4_ photosynthesis evolved at least 66 times in the last 35 million years in response to inefficiencies in C_3_ photosynthesis under hot, arid and low CO_2_ environments that promote photorespiration (Sage, 2016). In C_4_ plants, a CO_2_‐concentrating mechanism increases CO_2_ concentrations at the site of ribulose‐1,5‐bisphosphate carboxylase/oxygenase (RuBisCO) in bundle sheath cells, resulting in an increase in its carboxylase activity, a reduction in photorespiration and an increase in photosynthetic efficiency (Hatch, 1987). Compared with their C_3_ counterparts, C_4_ plants require one‐third of the amount of RuBisCO to achieve the same or higher rates of net CO_2_ fixation, leading to greater nitrogen‐use efficiency (Ghannoum *et al*., 2011). C_4_ plants also have higher water‐use efficiency because they can maintain a lower stomatal conductance relative to the rate of net photosynthetic CO_2_ assimilation (*A*), which supports greater biomass production when water is limited (Evans & von Caemmerer, 1996; Leegood, 2002; Taylor *et al*., 2010; Ghannoum *et al*., 2011). Thus, C_4_ plants tend to outperform C_3_ plants in hot and dry environments, lending them a competitive advantage in subtropical and semiarid ecosystems. As a result, C_4_ plants occupy *c*. 20% of the land surface and contribute up to 23% of global gross primary productivity, although they comprise < 5% of terrestrial plant species (Still *et al*., 2003; Luo *et al*., 2024).

Currently, C_4_ global primary productivity is typically estimated in Earth System Models using a simplified mechanistic model that uses parameters reflecting photosynthetic capacity (Still *et al*., 2019; Griffith *et al*., 2020). The model and its parameter values are largely derived from data collected on a single NADP‐dependent malic enzyme (NADP‐ME) subtype monocot, *Zea mays* (maize; Collatz *et al*., 1992). Consequently, the model does not capture substantial variation across C_4_ species in the biochemistry underlying CO_2_ assimilation (Hatch, 1987), or in their growth forms (Poorter *et al*., 2009; Liu *et al*., 2019). The responses of photosynthetic capacity to environmental parameters, such as temperature and irradiance, are also taken from the same study on maize (Collatz *et al*., 1992). This poses a problem because NADP‐ME subtype monocots (e.g. maize) only account for 31% of all C_4_ species and are predominantly found in regions with relatively high precipitation, due to their greater drought sensitivity than other C_4_ subtypes and growth forms (Ripley *et al*., 2010; Fan, 2023; Raubenheimer *et al*., 2023). Thus, it remains unclear whether data from a domesticated C_4_ NADP‐ME type species can reliably represent the photosynthetic performance of all C_4_ plants, including nondomesticated species and those belonging to other C_4_ subtypes. Over the last 30 yr, many studies have reported how C_4_ photosynthetic parameters vary in response to environmental drivers and described genetic differences within and between species. However, these findings are scattered across publications, except for an analysis by Pignon & Long (2020), and have not been subjected to data synthesis in a manner that could advance the representation of this important plant functional type in Earth System Models.

The most common way to estimate leaf‐level photosynthetic capacity is to measure CO_2_ response curves of net CO_2_ assimilation rate (i.e. *A*/*C*_i_ curves), and assess both the initial slope of the *A*/*C*_i_ curve (a parameter closely related to the apparent maximum activity of phosphoenolpyruvate (PEP) carboxylase, *V*_pmaxA_) and the horizontal asymptote of the *A*/*C*_i_ curve (i.e. the rate of CO_2_‐saturated net photosynthesis, termed *A*_max_). The initial slope of a C_4_ *A*/*C*_i_ curve is generally insensitive to changes in leaf measurement temperatures (*T*_leaf_) for plants grown at moderate temperatures (Long & Woolhouse, 1978; Laisk & Edwards, 1997; Sage, 2002), although it can be reduced in plants grown under chilling conditions, possibly reflecting an increase in the activation energy of PEP carboxylase (PEPc) at low temperatures (Pittermann & Sage, 2001; Kubien & Sage, 2004). By contrast, *A*_max_ increases with increasing *T*_leaf_ in C_4_ plants grown under moderate temperatures (Sage & Kubien, 2007). As with *T*_leaf_, the initial slope of a C_4_ *A*/*C*_i_ curve remains largely stable when measured at moderate vs high photosynthetic photon flux density (*PPFD*; e.g. 500 vs 1500 μmol photons m^−2^ s^−1^), suggesting that irradiance does not directly change PEPc kinetics, and PEP regeneration may not be the limiting step of photosynthesis at low *C*_i_ (Pengelly *et al*., 2010). However, *A*_max_ is sensitive to changes in irradiance and increases when measured under high *PPFD* (Pengelly *et al*., 2010). This irradiance response of *A*_max_ may result from the alleviation of chloroplastic electron transport limitation under high *PPFD* (Ermakova *et al*., 2019, 2023), thereby enhancing energy availability for PEP or ribulose 1,5‐bisphosphate (RuBP) regeneration, leading to higher overall net photosynthesis (von Caemmerer & Furbank, 2016). Taken together, the responses of photosynthetic capacity to measurement conditions are dynamic, with the initial slope of the *A*/*C*_i_ curve tending to be less affected by *T*_leaf_ and *PPFD* than *A*_max_.

C_4_ photosynthetic capacity can also be altered by growth temperatures. There is evidence that when measured under the same conditions, photosynthetic capacity (particularly *A*_max_) is reduced in C_4_ plants grown at warm to high temperatures (i.e. 30–43°C) compared with control plants. This is resulted from lower RuBisCO content and/or activity, NADP‐ME activity, cytochrome *f* content and carbonic anhydrase activity (Pearcy, 1977; Ward, 1987; Dwyer *et al*., 2007). By contrast, the activity of PEPc (and potentially *V*_pmaxA_) may not be affected by high growth temperatures, possibly due to its high thermal stability (Chen *et al*., 1994; Chinthapalli *et al*., 2002; Boyd *et al*., 2015). At low growth temperatures, many chilling‐tolerant, cool‐adapted C_4_ plants show no change in photosynthetic rates or RuBisCO concentrations compared with control plants (Pittermann & Sage, 2001; Cavaco *et al*., 2003; Naidu *et al*., 2003; Kubien & Sage, 2004), although the content of other photosynthetic enzymes (e.g. pyruvate, phosphate dikinase (PPDK)) can increase (Wang *et al*., 2008). These findings highlight that while some C_4_ plants can adjust their photosynthetic machinery in response to growth temperature, both high and low temperatures can impose biochemical constraints that limit photosynthetic capacity via acclimation and thermal stress.

Given that C_4_ photosynthesis is thought to be an evolutionary response to high photorespiratory loads (Sage, 2004), there has been considerable interest in the response of C_4_ photosynthesis to variation in CO_2_ concentrations. When C_4_ plants are grown in controlled environments, *A*_max_ can increase under subambient CO_2_ conditions, reflecting an up‐regulation of RuBisCO capacity to enhance carbon capture (Ripley *et al*., 2013; Pinto *et al*., 2014; Cunniff *et al*., 2017), but remains largely unchanged under the elevated CO_2_ levels projected for the coming century (Leakey, 2009; Heckman *et al*., 2024). By contrast, while the capacity of PEPc in controlled environment‐grown plants can also be increased at glacial CO_2_ concentrations, it can be reduced at elevated CO_2_ concentrations (Wong, 1979; Ghannoum *et al*., 2000; Pinto *et al*., 2014). However, when C_4_ plants are grown in the field, changes in growth CO_2_, concentrations had little effect on photosynthesis or the activity/capacity of key photosynthetic enzymes including PEPc (Leakey *et al*., 2006; Leakey, 2009; Markelz *et al*., 2011). Both subambient and elevated CO_2_ concentrations influence stomatal conductance, leading to changes in plant water relations and interactions with drought stress that can be complex in nature and create opportunities for crop improvement (Ghannoum *et al*., 2000; Markelz *et al*., 2011; Leakey *et al*., 2019). Although our understanding of how C_4_ plants respond to changes in CO_2_ concentration and drought, along with their underlying physiological mechanisms, has advanced in recent decades (e.g. Tissue *et al*., 1995; Wang *et al*., 2020; Ding *et al*., 2022), this knowledge has yet to be fully integrated into ecosystem models (Cowling *et al*., 2007; Still *et al*., 2019).

There is some evidence suggesting that the response of C_4_ photosynthetic capacity to growth environment depends on the biochemistry, life history and plant functional type of the species (e.g. growth form: monocot vs eudicot;Liu *et al*., 2019). Based on the decarboxylases involved, the C_4_ photosynthetic pathway can be categorised into three classical biochemical subtypes: NADP‐ME subtype, NAD‐dependent malic enzyme (NAD‐ME) subtype and PEP‐carboxykinase (PCK) subtype (Hatch, 1987; von Caemmerer & Furbank, 2016). Considerable variation in biochemical components (e.g. enzyme abundance) and photosynthetic capacity has been found in C_4_ plants of different growth forms within and between C_4_ biochemical subtypes. For example, the maximum carboxylation activity of PEPc and RuBisCO, *A*_max_ and leakiness (i.e. the CO_2_ fraction that leaks out from bundle sheath cells) responded to a short‐term increase in *T*_leaf_ differently among the three C_4_ biochemical subtypes (Sonawane *et al*., 2017). In addition, C_4_ annual species have been shown to exhibit a greater sensitivity of the chloroplastic electron transport capacity to *T*_leaf_ than C_4_ perennial species (Smith & Dukes, 2017). These findings suggest that differences in C_4_ biochemistry and growth form could play a role in determining photosynthetic responses to environmental conditions.

To understand how C_4_ photosynthetic capacity responds to environmental cues – and whether these responses differ systematically among biochemical subtypes and growth forms – data are needed from diverse species grown and measured under contrasting environmental conditions. Unfortunately, while numerous studies have evaluated photosynthetic capacity of C_4_ plants under various growth and measurement conditions (reference herein), these data are largely siloed in their separate publications, making it challenging to identify broad patterns in photosynthetic traits across C_4_ species. In this study, we used published gas‐exchange data to explore the drivers of variation in C_4_ photosynthetic capacity. Here, *A*/*C*_i_ curves measured from diverse C_4_ species across studies were collated to estimate *V*_pmaxA_ and *A*_max_ with a widely used C_4_ mechanistic model (von Caemmerer & Furbank, 1999; von Caemmerer, 2000, 2021). We assessed how *V*_pmaxA_ and *A*_max_ are affected by species‐specific traits (biochemical subtype and growth form) and environmental parameters (growth and measurement conditions). For growth conditions, we considered growth temperature and growth CO_2_ concentration. We also considered growth location (whether plants were grown in outdoor fields or in indoor controlled environments; see the Materials and Methods section for definitions). Compared with outdoor plants, indoor plants may experience limited growth space and different environments (e.g. fewer pests, relatively steady temperature and light conditions), which can affect overall morphology and physiology (Poorter *et al*., 2016). For measurement conditions, we were interested in how C_4_ photosynthetic capacity varies with measurement *T*_leaf_ and *PPFD*. An *A*/*C*_i_ curve should be measured under light‐saturating conditions, such that *PPFD* is often not considered a key factor in photosynthetic capacity. However, the *PPFD* required for light saturation of net photosynthesis can vary with growth conditions, and *A*/*C*_i_ curves can also be measured under sub‐saturating *PPFD* to address specific research questions (e.g. Sonawane *et al*., 2018).

Using these data, we test the following key hypotheses:

1. *V*_pmaxA_ and *A*_max_ are positively correlated, and both vary systematically with C_4_ biochemical subtype and growth form.
2. Indoor plants show a higher photosynthetic capacity than their outdoor counterparts due to more optimal growth conditions.
3. Cool‐grown plants have a higher photosynthetic capacity relative to their warm‐grown counterparts when measured at a common *T*_leaf_. While *A*_max_ increases with increasing *T*_leaf_, *V*_pmaxA_ remains relatively stable.
4. Elevated growth CO_2_ concentrations have no effect on photosynthetic capacity in C_4_ species.

Lastly, we assess whether the photosynthetic capacity of popular C_4_ model species – maize, *Sorghum bicolor* (sorghum) and *Setaria viridis* – the three most abundant species in our dataset, is similar to that of other C_4_ species to assess whether data from these three species can be used to represent the broad range of undomesticated C_4_ species found in nature. To date, no study has explicitly tested whether model C_4_ species differ in photosynthetic capacity from non‐model species using a comprehensive dataset.

## Materials and Methods

### Data acquisition

This work was conceived by members of the C_4_ Working Group supported by the US Geological Survey's Powell Center, with data collaboratively sourced within the group and from researchers closely associated with its members. C_4_ *A*/*C*_i_ data were compiled from 52 studies published between 2001 and 2024 (Anderson *et al*., 2001; Pittermann & Sage, 2001; Cousins & Bloom, 2003; Kim *et al*., 2006; Leakey *et al*., 2006; Nippert *et al*., 2007; Ripley *et al*., 2007, 2008, 2010, 2013; Carmo‐Silva *et al*., 2008; Cunniff *et al*., 2008; Horst *et al*., 2008; Mantlana *et al*., 2008; Osborne *et al*., 2008; Soares *et al*., 2008; Kakani *et al*., 2008a,b; Pengelly *et al*., 2010; Arena *et al*., 2011; de Souza, 2011; Pinto *et al*., 2011, 2014; Soares‐Cordeiro *et al*., 2011; Bloom *et al*., 2012; Fay *et al*., 2012; Feng *et al*., 2012; Sun *et al*., 2012; Vogan & Sage, 2012; Wang *et al*., 2012; Chen *et al*., 2013; Sage *et al*., 2013; Bissinger *et al*., 2014; Friesen *et al*., 2014; Ge *et al*., 2014; Oakley *et al*., 2014; Sharwood *et al*., 2014; Xu *et al*., 2014; Głowacka *et al*., 2015; Peixoto & Sage, 2017; Sonawane *et al*., 2017, 2018; Smith & Dukes, 2018; Li *et al*., 2019, 2021, 2022; Khoshravesh *et al*., 2020; Pathare *et al*., 2020; Danila *et al*., 2021; Peixoto *et al*., 2021; Liu *et al*., 2023; Ermakova *et al*., 2024; Gan & Sage, 2024) and five unpublished datasets with permission to use. Together, 1696 C_4_ *A*/*C*_i_ curves for 74 species of 12 plant families from 57 unique studies were included in the analysis. Raw data files (i.e. direct output files from the measuring instrument) were requested from authors where available. In cases where the raw data files were not available, curves were digitised from published figures as per Pignon & Long (2020). The compiled dataset is provided in Supporting Information Dataset S1.

### *A*/*C*_i_ curve analysis

To fit the curves and estimate parameters, the 1696 individual *A*/*C*_i_ curves were consolidated into 543 groups. Replicate measurements of the same species and genotype that were measured within 5 d in a study were considered as one experimental group. This approach allowed us to minimise software crashes due to a lack of model convergence and estimate sampling variance; this grouping approach has been used in other studies (e.g. Wu *et al*., 2024). The *A*/*C*_i_ data at *C*_i_ < 100 μmol mol^−1^ (i.e. the initial slope of the *A*/*C*_i_ curve) were used to estimate *V*_pmaxA_, according to von Caemmerer (2000):

(Eqn 1) $$A=V_{p}-R_{m}=\frac{C_{i}V_{\text{pmaxA}}}{C_{i}+K_{p}}-R_{m}$$

where *A* is the net CO_2_ assimilation rate (μmol CO_2_ m^−2^ s^−1^), *V*_p_ is the rate of PEP carboxylation (μmol CO_2_ m^−2^ s^−1^), *C*_i_ is the CO_2_ concentration in the intercellular space (μmol mol^−1^ CO_2_), *K*_p_ is the Michaelis–Menten constant for CO_2_ (μ bar; converted to concentrations using a solubility for CO_2_ of 0.0334 mol bar^−1^ and atmospheric pressure at the measurement site; see von Caemmerer *et al*. (1994)), and *R*_m_ is daytime mitochondrial respiration in mesophyll cells (μmol CO_2_ m^−2^ s^−1^). Under low *C*_i_, *A* is linearly correlated with the maximum PEPc activity in mesophyll cells, given that the leakage of CO_2_ from bundle sheath cells is low and is generally ignored (von Caemmerer, 2021). We are aware that leakage of CO_2_ from bundle sheath cells could increase when measurements are done at low light (i.e. *PPFD* < 100 μ mol photons m^−2^ s^−1^; Yin *et al*., 2016). Given that our dataset contains no measurements below *PPFD* = 100 μmol photons m^−2^ s^−1^, we consider the potential impact of CO_2_ leakage on our estimates of *V*_pmaxA_ to be minimal. We also assumed that PEP substrate concentrations were saturating under the measurement condition, and *A* was not limited by PEP regeneration, as required by the C_4_ model (von Caemmerer, 2000, 2021). *K*_p_ was adjusted to account for variation in *T*_leaf_ according to Boyd *et al*. (2015):

(Eqn 2) $$K_{p}=K_{p25}\times e^{E_{a}\left(T_{\text{leaf}}-25\right)/\left(298.15R\left(T_{\text{leaf}}+273.15\right)\right)}$$

where *K*_p25_ is *K*_p_ measured at 25°C (assumed to be 80 μ bar; Bauwe, 1986; DiMario & Cousins, 2019), *E*_a_ is the activation energy of *K*_p_ (36.3 kJ mol^−1^; Boyd *et al*., 2015), and *R* is the molar gas constant (0.008314 kJ K^−1^ mol^−1^). *R*_m_ was taken as half the daytime leaf mitochondrial respiration rate (*R*_day_; μmol CO_2_ m^−2^ s^−1^; von Caemmerer, 2000). Given that there are few *R*_day_ measurements in C_4_ plants, *R*_day_ was assumed to equal leaf dark respiration such that *R*_day_ = 1.2 μmol CO_2_ m^−2^ s^−1^ at 25°C (termed as *R*_day25_), according to a data synthesis of dark respiration in 39 C_4_ species (Fan *et al*., 2022). We chose not to scale dark respiration with photosynthetic capacity (i.e. *A*_max_) across all C_4_ species, as the commonly used scaling relationship was derived from maize (Collatz *et al*., 1992). Applying this maize‐based relationship would undermine our objective of comparing model species (including maize) with non‐model species. *R*_day_ was adjusted for variation in *T*_leaf_ using a *Q*_10_ equation (Atkin & Tjoelker, 2003):

(Eqn 3) $$R_{\mathrm{day}}=R_{\mathrm{day}25}\times Q_{10}^{\frac{T_{\text{leaf}}-25}{10}}$$

where *Q*_10_ is 2 (Huntingford *et al*., 2013).

Although mechanistic models for enzyme‐ and light‐limited C_4_ photosynthesis are available, C_4_ photosynthesis could be co‐limited by RuBisCO activity, RuBP regeneration and/or PEP regeneration at high *C*_i_, with all three processes requiring energy from light (von Caemmerer & Furbank, 1999). Thus, as no information was provided on the limitation status of photosynthesis at high *C*_i_ by individual studies, we estimated *A*_max_ using the entire *A*/*C*_i_ curve. The *A*_max_ was estimated by the horizontal asymptote of a four‐parameter non‐rectangular hyperbolic function (Leakey *et al*., 2006; Li *et al*., 2022):

(Eqn 4) $$\theta {\left(A+R_{\mathrm{day}}\right)}^{2}-\left(\alpha C_{i}+A_{\max}\right)\left(A+R_{\mathrm{day}}\right)+\alpha C_{i}A_{\max}=0$$

where θ is the curvature factor of an *A*/*C*_i_ curve (unitless), which was assumed to be 0.7 for C_4_ plants (Sonawane *et al*., 2018; von Caemmerer, 2021), and α is the initial slope of an *A*/*C*_i_ curve (μmol CO_2_ m^−2^ s^−1^). We did not include mesophyll conductance as a term in Eqn (4) due to a lack of such data in examined studies. However, by assuming θ = 0.7, we considered a potential drop in the *C*_i_ between the intercellular space and the site of RuBisCO carboxylation (i.e. bundle sheath cells; Evans, 1989). All curve fitting was performed using the non‐linear least square (nls) function and sampling variances of every fit were extracted using the vcov function in R (v.4.4.1, R Core Team, 2024). The number of individual curves per group (*n*_group_, ranging from 1 to 9) was also reported. Sampling variance of parameters (i.e. squared SE for parameters) was subsequently considered in the statistical models (Nakagawa & Santos, 2012; Noble *et al*., 2017, 2022).

### Data classification and summary

Among the 543 groups, eight groups contained fewer than two data points at low *C*_i_ (i.e. < 100 μmol mol^−1^), while 131 groups contained fewer than two data points at high *C*_i_ (i.e. > 500 μmol mol^−1^). As this lack of data is likely to influence the accuracy of model fitting using these portions of the *A*/*C*_i_ curve, the corresponding estimated *V*_pmaxA_ and *A*_max_ values of these groups were discarded. Given that measurements within a single group were made under the same measurement temperature and irradiance, we provided averaged values of *T*_leaf_ and *PPFD* per group. We further assessed the estimated parameters of each group: if the curve violated the assumption that *A* is limited by *V*_pmaxA_ at low *C*_i_ (as indicated by *V*_pmaxA_‐limited photosynthesis being higher than *A*_max_‐limited photosynthesis at low *C*_i_), this group of *A*/*C*_i_ curves was discarded as per Pignon & Long (2020). This assessment led to 108 additional groups being removed from the final dataset (Dataset S2), many of which included plants subjected to severe abiotic stresses, such as chilling, drought and low nutrients.

**Fig. 1:**
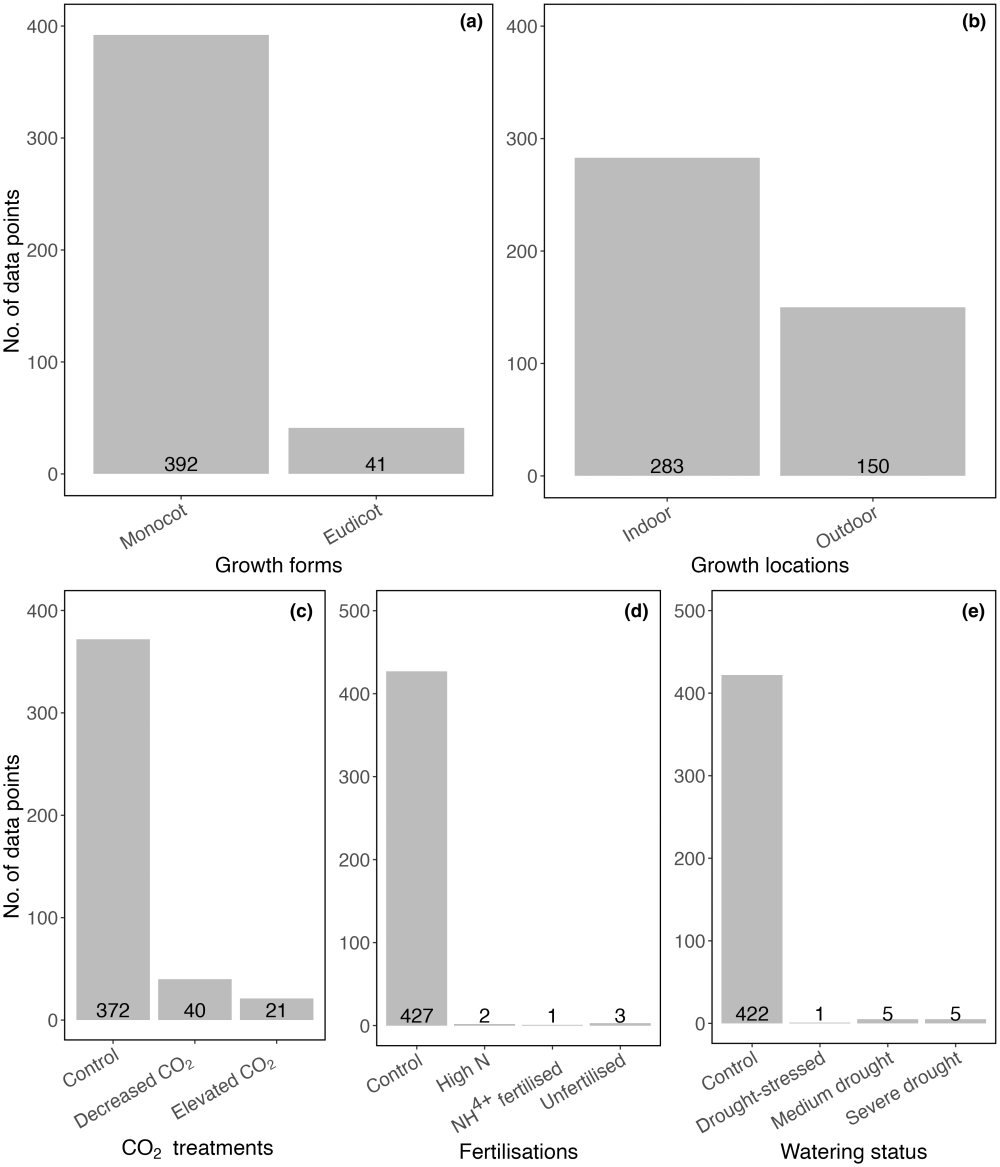
Summary of data points (by groups) based on (a) growth forms, (b) locations, (c) CO_2_ treatments, (d) fertilisation treatments and (e) watering status. Decreased and elevated CO_2_ treatments consist of measurements made on plants with a growth CO_2_ concentration of < 400 and > 400 ppm, respectively. See the Materials and Methods section for more information.

The remaining measurements were classified according to their C_4_ biochemical subtype (NADP‐ME, NAD‐ME or PCK subtype), growth form (monocot or eudicot) and growth location (indoor or outdoor). Indoor plants refer to those grown in pots within controlled environment chambers or glasshouses. By contrast, outdoor plants are grown directly in the soil or in common gardens without pot restrictions. Experiments conducted at Free‐Air Carbon Dioxide Enrichment (FACE) and Lysimeter CO_2_ Gradient (LYCOG) facilities are classified as outdoor‐grown, as plants in these settings are planted directly in soil. The growth CO_2_ concentration was noted, where the ambient CO_2_ level was assumed to be 400 ppm if not given by the study, as 400 ppm represents the average atmospheric CO_2_ concentration from 2001 to 2024 (Friedlingstein *et al*., 2025), which covers the period of publications used in this study, although the experiments themselves may have been conducted 1 or 2 yr earlier. For indoor‐grown plants, mean maximum (*T*_max_) and minimum (*T*_min_) growth temperatures were the set daytime and night‐time temperatures, respectively. We used the reported measurement *T*_leaf_ in our analyses, rather than normalising to a rate at a set temperature of 25°C, as we did not want to assume that the *V*_pmax_ and *A*_max_ of all species and growth environments will have the same sensitivity to short‐term changes in *T*_leaf_. For outdoor‐grown plants, mean *T*_max_ and *T*_min_ were the mean maximum and minimum temperatures observed across the experimental periods at the study sites, respectively. Growth *PPFD* was unavailable in most outdoor studies and was therefore excluded from the analysis. We were unable to consider either fertilisation treatment or water status as potential categories for analysis, as low nutrient treatment and/or water‐stressed conditions constituted < 5% of the data (Fig. 1d,e). A summary of measurements based on plant traits and growth treatments is given in Fig. 1.

Across the dataset, maize, sorghum and *S. viridis* were the three most commonly measured species, accounting for 18, 6 and 4% of the total measurements, respectively. Indeed, maize contributed 43% of the outdoor data and played an important role in the growth location category (Fig. S1). This species skewness is later accounted for in the statistical models (to be described later).

### Statistical analysis

To determine how different plant traits and experimental conditions altered *V*_pmaxA_ and *A*_max_, we ran two multivariate linear mixed‐effects models with *V*_pmaxA_ or *A*_max_ as the dependent variable in each model. These models accounted for the sampling variance of each parameter, with parameters having greater precision being weighted more heavily to overall means. We included three main fixed effect categories in our models: (1) species‐specific traits, including C_4_ subtype and growth form; (2) environmental growth conditions, such as growth location, temperature and CO_2_ concentration; and (3) measurement conditions, such as *T*_leaf_ and *PPFD*. Given that mean *T*_max_ and *T*_min_ were strongly positively correlated with each other (Fig. S2), we used mean *T*_max_ in the model. A list of fixed effects is given in Table 1.

**Table 1:** Results of multivariate linear mixed‐effects models testing the response of *V*_pmaxA_ and *A*_max_ against fixed effects of species‐specific traits and experimental conditions (see main text for more details).

	*V*_pmaxA_					*A*_max_				
	Estimate	SE	Lower CI	Higher CI	*P* value	Estimate	SE	Lower CI	Higher CI	*P* value
Fixed effects										
C_4_ subtype	−1.299	7.716	−16.470	13.873	0.969	−1.339	3.363	−7.960	5.282	0.727
Growth form	0.270	8.272	−15.996	16.535	0.196	−15.091	11.082	−37.636	7.455	0.295
Growth location	−15.190	5.154	−25.747	−4.634	0.005**	−6.676	2.651	−12.295	−1.057	0.018*
Mean *T*_max_	−2.549	1.362	−5.228	0.130	0.001**	0.367	0.754	−1.117	1.851	0.001**
Growth CO_2_	−0.018	0.012	−0.042	0.006	0.138	−0.003	0.009	−0.021	0.016	0.761
*T*_leaf_	3.580	0.849	1.910	5.250	< 0.0001***	2.406	0.476	1.468	3.343	< 0.0001***
*PPFD*	0.014	0.002	0.010	0.018	< 0.0001***	0.010	0.001	0.008	0.013	< 0.0001***
C_4_ subtype × *T*_leaf_	0.544	0.519	−0.477	1.565	0.504	0.187	0.261	−0.327	0.700	0.761
C_4_ subtype × Mean *T*_max_	−0.413	2.268	−4.873	4.047	0.840	−0.943	1.188	−3.283	1.396	0.642
C_4_ subtype × *PPFD*	−0.001	0.005	−0.010	0.008	0.674	0.002	0.003	−0.003	0.007	0.750
Growth form × *T*_leaf_	−1.412	0.982	−3.342	0.518	0.146	3.590	2.519	−1.368	8.549	0.219
Growth form × Mean *T*_max_	3.477	2.132	−0.890	7.845	0.099	0.148	1.207	−2.410	2.707	0.781
Growth form × *PPFD*	0.004	0.008	0.631	−0.012	0.645	−0.023	0.019	−0.066	0.007	0.143
*T*_leaf_ × Mean *T*_max_	0.013	0.036	−0.058	0.083	0.724	−0.043	0.020	−0.082	−0.004	0.033*
Species group	8.209	6.740	−5.385	21.802	0.230	3.467	2.381	−1.376	8.311	0.155
Overall model statistics										
Effect size/sample size	49/401					37/297				
Marginal *R* ^2^	0.51					0.47				
Conditional *R* ^2^	0.83					0.75				

Effect size denotes the number of studies, while the sample size is the number of *V*_pmaxA_ or *A*_max_ data points. Model's marginal *R* ^2^ reflects the variance explained by fixed effects only, whereas conditional *R* ^2^ considers the variance explained by both fixed and random effects. Bold text indicates statistical significance. Significant codes: *, *P* < 0.05; **, *P* < 0.01; ***, *P* < 0.001. Continuous variables (i.e. mean *T*_max_, growth CO_2_, *T*_*l*eaf_ and *PPFD*) were mean‐centred before the analysis. Lower and high CI: lower and upper 95% confidence intervals.

In addition, we included six *a priori* interaction terms examining the interactive effects of species‐specific traits, temperatures and irradiance: C_4_ subtype × mean *T*_max_, C_4_ subtype × *T*_leaf_, C_4_ subtype × *PPFD*, growth form × mean *T*_max_, growth form × *T*_leaf_ and growth form × *PPFD*. These terms were designed to test the question of whether the response of C_4_ photosynthetic capacity traits to short‐ and long‐term temperatures and measurement irradiance differ among C_4_ subtypes or growth forms. We confirmed that our *V*_pmaxA_ and *A*_max_ data per C_4_ subtypes and growth form were distributed across the entire range of mean *T*_max_, *T*_leaf_ and *PPFD* (Fig. S3), such that any significant interaction terms would not be biased due to separated data distributions. Data distribution with respect to mean *T*_max_ and *T*_leaf_ is shown in Fig. S4. Random effects included in the models were the different studies (indicated by publications), grouping of the curves (as described in the section *A*/*C*_i_ curve analysis) and species. Given that C_4_ model species (maize, sorghum and *S. viridis*) made up a significant proportion of the data, we also conducted a case study examining to what extent the model results obtained from data of all species match with those of the C_4_ model species and visualise our results with consideration of C_4_ model species (see the Results section).

The multivariate linear mixed‐effects models with restricted maximum likelihood estimation were run using the package metafor v.4.6.0 (Viechtbauer, 2010), and plots were made using the package ggplot2 v.3.5.1 (Wickham, 2016) and orchard v.2.0 (Nakagawa *et al*., 2021, 2023) on R program v.4.4.1 (R Core Team, 2024). Estimated effects were considered significant if *P* < 0.05 in the models.

## Results

### Positive correlations between *V*_pmaxA_ and *A*_max_

**Fig. 2:**
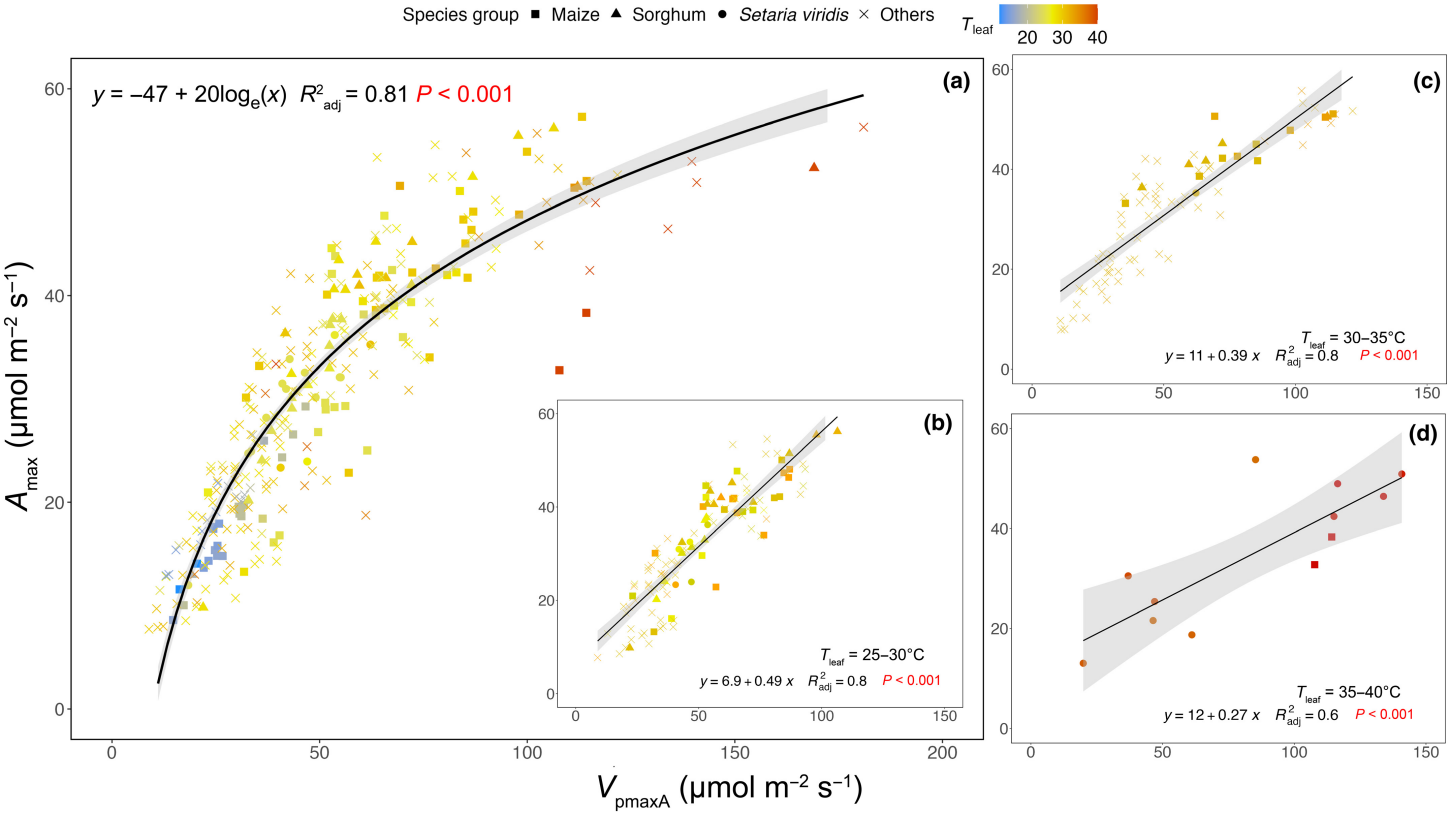
Correlation between *A*_max_ and *V*_pmaxA_ estimated from data collected from a range of species measured at various growth and measuring conditions. Point shapes reflect important species groups, and colours denote measurement temperature (*T*_leaf_). (a) All available *A*_max_‐*V*_pmaxA_ data; (b–d) data subsets measured at 25–30°C, 30–35°C and 35–40°C, respectively. Equation, adjusted *R* ^2^ values and *P* values of individual fits are shown in each panel. Shaded areas represent 95% confidence intervals around predicted means from the fitted curves.

In general, *V*_pmaxA_ and *A*_max_ were correlated with each other in a logarithmic manner (*R* ^2^ = 0.81; Fig. 2a). We found that for leaves measured at a moderate *T*_leaf_ (i.e. < 35°C), changes in *V*_pmaxA_ were tightly coupled with changes in *A*_max_ in a nearly linear fashion (Fig. 2a). When leaves were measured at a hotter *T*_leaf_ (i.e. > 35°C), the coupling between *V*_pmaxA_ and *A*_max_ broke down, and an increase in *V*_pmaxA_ was accompanied by a lesser increase in *A*_max_. This result was supported by significantly different slopes (*P* < 0.001; standardised major axis analysis) in the linear correlations between *V*_pmaxA_ and *A*_max_ for leaves measured at 25–30°C (slope = 0.49; Fig. 2b), 30–35°C (slope = 0.39; Fig. 2c) and 35–40°C (slope = 0.27; Fig. 2d). Together, these findings highlight that at high *A*_max_ (i.e. high C_i_), photosynthesis was less dependent on PEP carboxylation compared with other high‐temperature sensitive biochemical processes (e.g. RuBisCO carboxylation), or photosynthesis was constrained by increased limitations in light and bundle sheath (CO_2_) at high *A*_max_ (e.g. affecting RuBP regeneration).

### Factors influencing *V*_pmaxA_ and *A*_max_ are mostly environmental

**Fig. 3:**
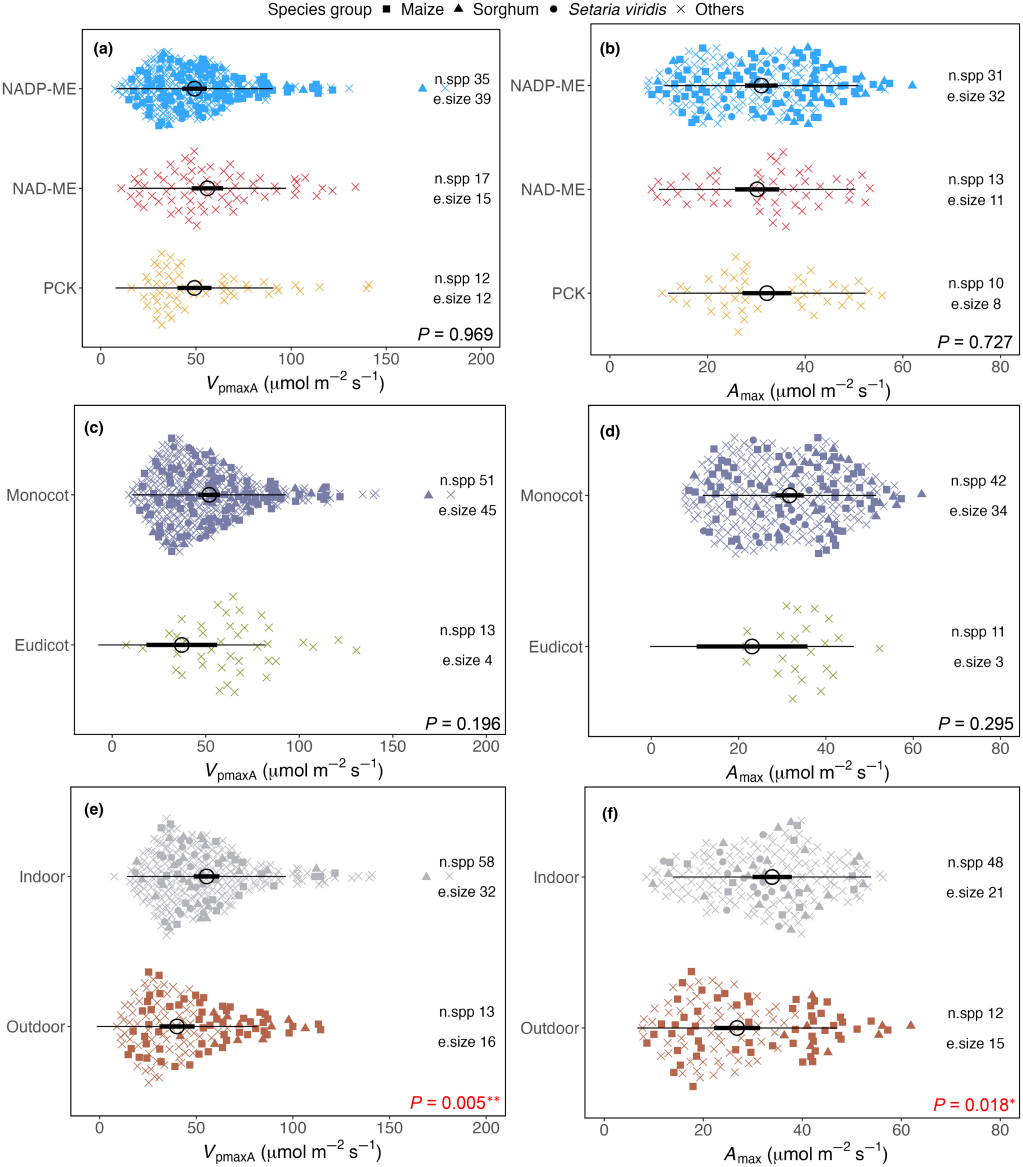
*V*_pmaxA_ and *A*_max_ plotted by C_4_ biochemical subtypes (a and b, respectively), growth forms (c and d, respectively) and growth locations (e and f, respectively). Data presented here are a mixture of measurements done at various growth and measuring temperatures and irradiance. Raw data points are plotted as coloured symbols, with different symbol shapes reflecting important species groups. Model‐predicted marginal means and intervals are shown as a horizontal line. On this line, black circles indicate model‐predicted meta‐analytic means of *V*_pmaxA_ or *A*_max_; thick bars are 95% confidence intervals, and thin bars are 95% prediction intervals. On the right‐hand side of each panel, the number of unique species per category (n.spp) and the number of individual studies (e.size) are indicated. *P* values of the multivariate linear mixed‐effects model are indicated in each panel (see Table 1). Significant codes: *, *P* < 0.05; **, *P* < 0.01.

Multivariate linear mixed‐effects models highlighted several factors that significantly affected both *V*_pmaxA_ and *A*_max_ (Table 1). C_4_ subtype identity and growth form had little influence on values of either *V*_pmaxA_ or *A*_max_ (Fig. 3a–d). Note that the number of studies examining eudicots or PCK‐type plants was noticeably lower than studies focusing on monocots or NADP‐ME plants, respectively. This highlights an urgent need to further investigate photosynthetic characteristics in eudicots and PCK‐type plants. There is no evidence that *V*_pmaxA_ and *A*_max_ vary among families or subfamilies (*P* = 0.73 and 0.88 for *V*_pmaxA_ and *A*_max_, respectively) or within each biochemical subtype (Fig. S5).

**Fig. 4:**
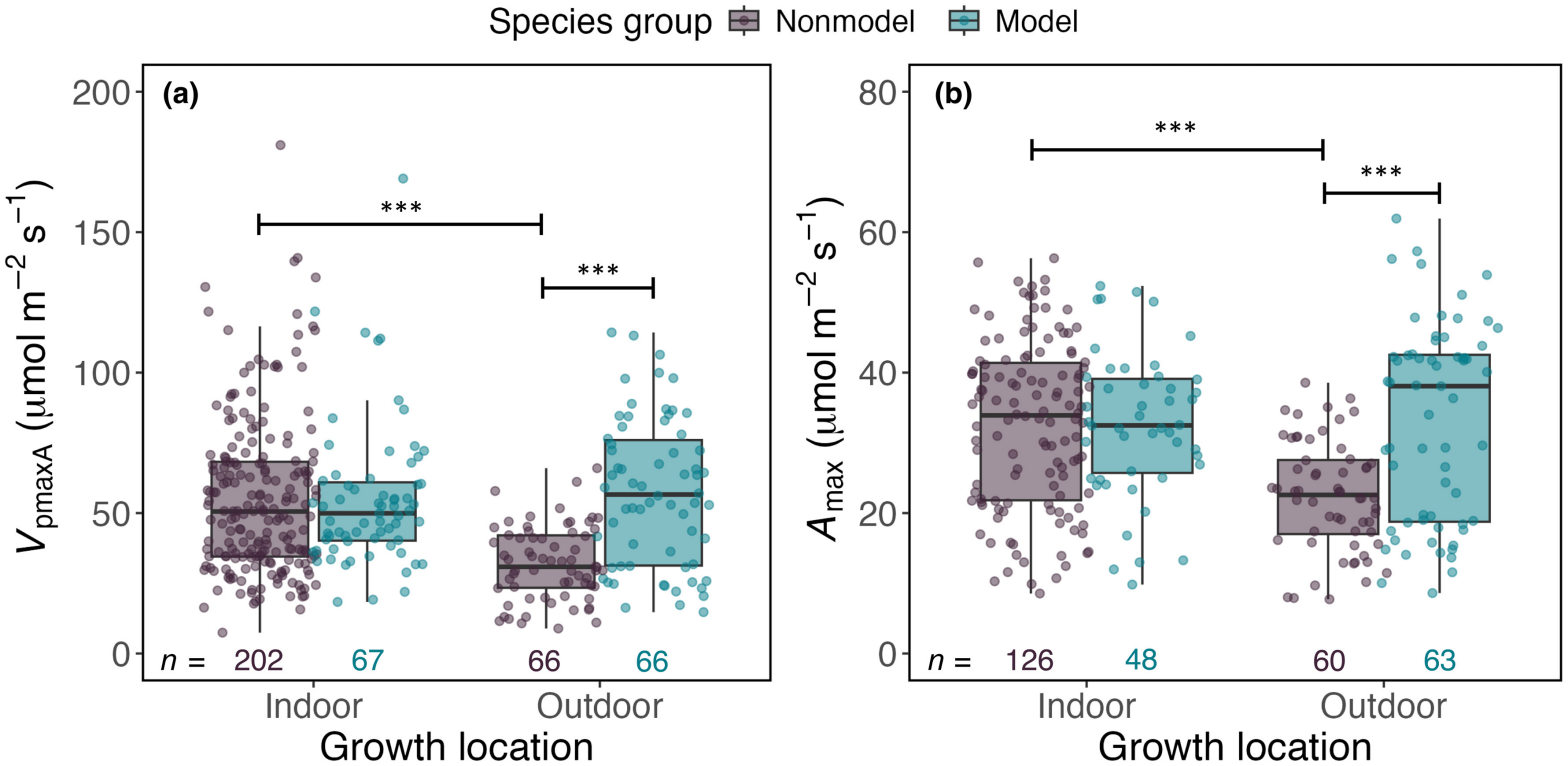
Comparison between *V*_pmaxA_ and *A*_max_ with growth location coloured by species group (C_4_ non‐model species vs model species). A linear mixed‐effect model was conducted to examine *V*_pmaxA_ and *A*_max_ between two species groups at a location, or within a species group at both locations. Statistical results of comparisons are denoted with horizontal lines (i.e. the two bars at the beginning and the end of a horizontal line are compared) and asterisks indicate statistical significance (***, *P* < 0.001) from this lnear mixed‐effect model. The sample size (*n*) for each group is indicated on plots. Boxplots show the median (horizontal line within each box), interquartile range (IQR; box edges spanning the 25^th^ to 75^th^ percentiles), and whiskers (1.5 × IQR).

We found that both *V*_pmaxA_ and *A*_max_ were higher when plants were grown indoors than in the field (*P* = 0.005 and 0.018 for *V*_pmaxA_ and *A*_max_, respectively; Fig. 3e,f), where averaged mean *T*_max_ values were 28.2 and 25.2°C, respectively, for indoor and field plants. Specifically, the models estimated that *V*_pmaxA_ of indoor plants was 35% higher than their outdoor counterparts (55.3 ± 1.7 vs 40.0 ± 2.1 μmol m^−2^ s^−1^), while *A*_max_ of indoor‐grown plants was 22% higher than those grown outdoors (33.9 ± 0.9 vs 26.8 ± 1.1 μmol m^−2^ s^−1^). It is worth noting that C_4_ model species (i.e. maize and sorghum) appear to be at the high‐end of the *V*_pmaxA_ and *A*_max_ spectra, particularly for outdoor‐grown plants (Fig. 3e,f). We thus further examined whether there was an interaction between species group and growth location, and found it to be significant (*P* < 0.001). The mean values of *V*_pmaxA_ and *A*_max_ were significantly higher in outdoor‐grown C_4_ model species than in non‐model species grown outside (*P* < 0.001; Fig. 4). This result is further supported by comparisons of the photosynthetic capacity of maize and sorghum against other species (Fig. S6). However, no significant differences were observed between model and non‐model species grown indoors (Figs 4, S6).

**Fig. 5:**
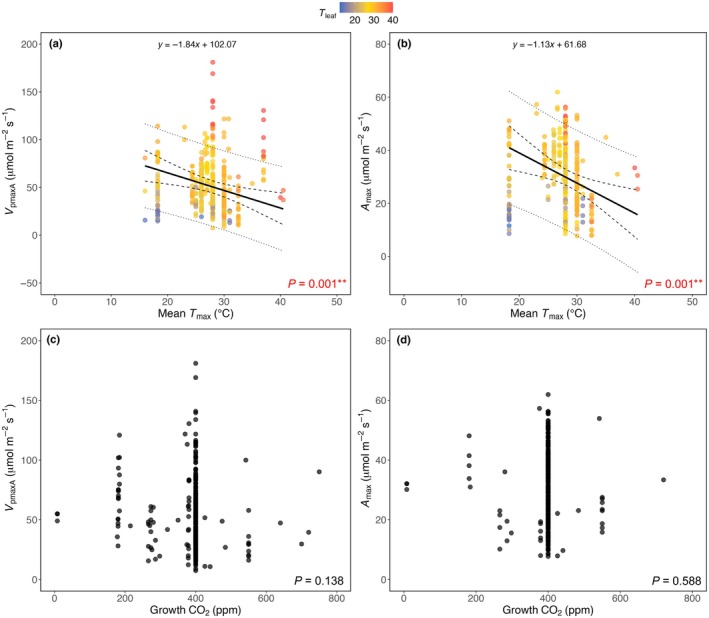
Relationships between *V*_pmaxA_ and *A*_max_ with mean *T*_max_ (a and b, respectively) and growth CO_2_ levels (c and d, respectively). Data points in (a) and (b) are coloured in a gradient by the measurement temperature (*T*_leaf_). Solid lines represent model‐predicted values of *V*_pmaxA_ or *A*_max_ at a given mean *T*_max_ (equations are shown at the top of each panel), dashed lines indicate 95% confidence intervals, and dotted lines show 95% prediction intervals. *P* values of the multivariate linear mixed‐effects models are indicated. Significant codes: **, *P* < 0.01.

**Fig. 6:**
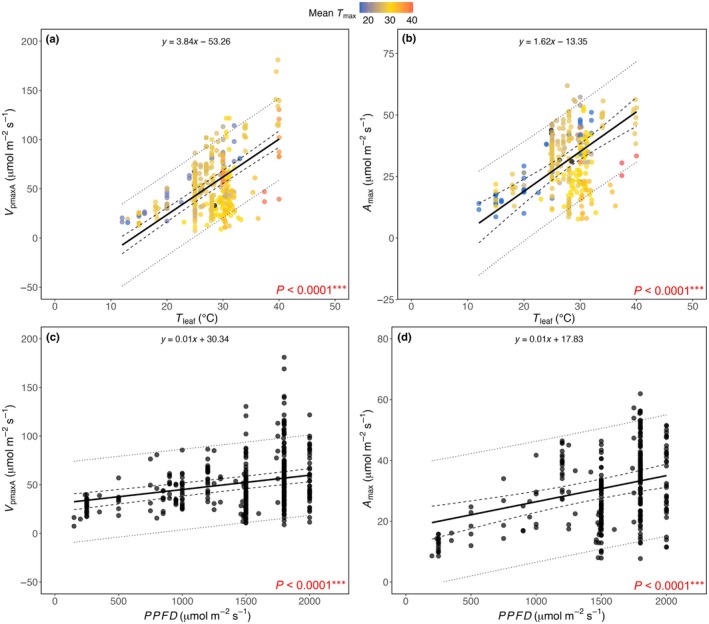
Relationships between *V*_pmaxA_ and *A*_max_ with *T*_leaf_ (a and b, respectively) and photosynthetic photon flux density (*PPFD*) (c and d, respectively). Data points in (a) and (b) are coloured by the mean maximum growth temperature (mean *T*_max_). Solid lines represent model‐predicted values of *V*_pmaxA_ or *A*_max_ at a given *T*_leaf_ or *PPFD* (equations are shown at the top of each panel), dashed lines indicate 95% confidence intervals, and dotted lines show 95% prediction intervals. *P* values of the multivariate linear mixed‐effects models are indicated. Significant codes: ***, *P* < 0.001.

We also found that *V*_pmaxA_ and *A*_max_ decreased significantly with increasing mean *T*_max_ (*P* = 0.001; Fig. 5a,b), with this pattern being stronger for a subset of data measured at a *T*_leaf_ between 25 and 30°C to account for variation in *T*_leaf_ in the analysis (Fig. S7). No effect of growth CO_2_ concentrations was found on either *V*_pmaxA_ or *A*_max_ (Fig. 5c,d). In contrast to the negative response to mean *T*_max_, *V*_pmaxA_ and *A*_max_ increased significantly with increasing *T*_leaf_ (*P* < 0.0001; Fig. 6a,b) and *PPFD* (*P* < 0.0001; Fig. 6c,d). These results highlight the different responses of *V*_pmaxA_ and *A*_max_ to short‐term changes in *T*_leaf_ and long‐term acclimation to mean *T*_max_ (to be described later). To assess whether the differences in *V*_pmaxA_ and *A*_max_ between growth locations (Fig. 3e,f) were linked to the distinct growth and measurement conditions experienced by indoor and outdoor plants, we compared the slopes of their correlations with mean *T*_max_ (Fig. S8), growth CO_2_ concentration (Fig. S9), *T*_leaf_ (Fig. S10) and *PPFD* (Fig. S11) separately for each location (Table S1). We found that, apart from the *V*_pmaxA_‐*T*_leaf_ correlation, which differed significantly between growth locations (*P* = 0.003), there was no evidence that growth location explained the effects of mean *T*_max_ or *T*_leaf_ on *V*_pmaxA_ or *A*_max_ (Table S2). We further examined whether different C_4_ subtypes or growth forms varied in their response to mean *T*_max_, *T*_leaf_ and *PPFD* by considering interaction effects. None of the interaction terms was significant (Table 1), suggesting that the responses of *V*_pmaxA_ and *A*_max_ to growth and measuring temperatures and irradiance are independent of species‐specific traits (such as growth form). Overall, the fixed effects considered in our models accounted for 51 and 47% of the variation in *V*_pmaxA_ and *A*_max_, respectively (Table 1).

### Interactive effects of *T*_leaf_ and mean *T*_max_ on photosynthetic capacity

**Fig. 7:**
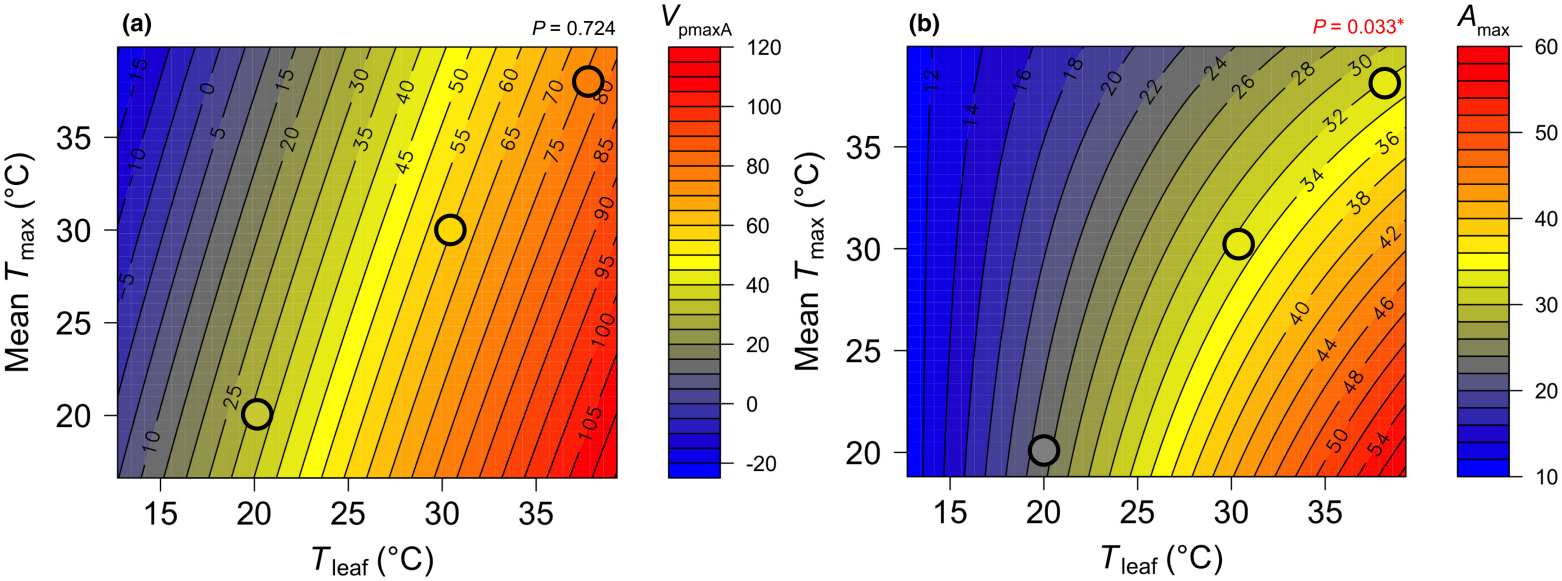
Contour plots illustrating the model‐predicted responses of (a) *V*_pmaxA_ and (b) *A*_max_ (μmol m^−2^ s^−1^) to *T*_leaf_ and mean *T*_max_. *P* values of linear mixed‐effects model testing the interaction between *T*_leaf_ and mean *T*_max_ are indicated (see Table 1). Black circles represent comparisons of *V*_pmaxA_ and *A*_max_ at the same mean *T*_max_ and *T*_leaf_ of 20°C, 30°C and 38°C, with the colour gradient reflecting changes in *V*_pmaxA_ and *A*_max_ (see the colour legend on the right‐hand side of each panel). Significant codes: *, *P* < 0.05.

The different responses of *V*_pmaxA_ and *A*_max_ to changes in long‐term mean *T*_max_ and short‐term *T*_leaf_ pointed towards a possible interaction between these two temperature factors (Figs 5, 6). We thus explored the mean *T*_max_ × *T*_leaf_ interaction and the result showed that the *V*_pmaxA_ was not affected by the interaction between growth and measurement temperatures (*P* = 0.724; Table 1), although the interaction term was significant for *A*_max_ (*P* = 0.033). We further illustrated the complex responses of *V*_pmaxA_ and *A*_max_ to mean *T*_max_ and *T*_leaf_ using contour plots (Fig. 7). The contour plots show that at any given mean *T*_max_, the sensitivity of *V*_pmaxA_ to changes in *T*_leaf_ was not influenced by acclimation to different mean *T*_max_ (Fig. 7a). This pattern also means that *V*_pmaxA_ measured at the predominant leaf temperatures of warm‐grown plants is higher than *V*_pmaxA_ measured at the predominant leaf temperatures of plants grown at lower temperatures. *A*_max_ also increased with increasing *T*_leaf_, but the increase in *A*_max_ per 1°C increase in *T*_leaf_ was higher in plants grown in cooler environments (i.e. mean *T*_max_ < 25°C) than in warmer environments (Fig. 7b). This suggests that *A*_max_ was more sensitive to changes in *T*_leaf_ in plants acclimated to lower mean *T*_max_. This pattern highlights that while *A*_max_ is lower in plants grown and measured at 20°C, compared with those grown and measured at 30°C and 38°C, *A*_max_ in plants grown and measured at 30°C and 38°C is similar (Fig. 7b).

### Impact of C_4_ model species on the analysis

Maize, sorghum and *S. viridis*, three frequently studied species in C_4_ research, dominated our *A*/*C*_i_ dataset (Fig. S1). In fact, data collected from these C_4_ model species are widely used to model the response of C_4_ non‐model species at the ecosystem level (e.g. in the Community Land Model (Lawrence *et al*., 2019)). Consequently, we investigated whether *V*_pmaxA_ and *A*_max_ of these C_4_ model species are representative of the non‐model species. We found no significant difference in *V*_pmaxA_ and *A*_max_ between the C_4_ model and non‐model species (*P* = 0.230 and 0.155 for *V*_pmaxA_ and *A*_max_, respectively; Table 1 – species group). However, it is worth noting that the interaction between species group and growth location was significant due to the lower photosynthetic capacity of non‐model species grown outdoors (as described in the previous section and Fig. 4). Therefore, we suggest that while our results show that C_4_ model species have similar photosynthetic capacity compared with non‐model species, care must be taken when extending this finding to natural outdoor settings, the conditions of most interest to ecologists and Earth System Model modellers. Lastly, our results indicate that our multivariate model was not biased by the weighting of data from C_4_ model species.

## Discussion

This study aimed to explore general patterns of C_4_ photosynthetic capacity, indicated by *V*_pmaxA_ and *A*_max_, among a wide range of species grown and measured at various conditions. We found that *V*_pmaxA_ and *A*_max_ were tightly coupled in a way that reflects the limitation states of C_4_ photosynthesis at different *C*_i_ and *T*_leaf_ conditions, partially supporting Hypothesis 1 (Fig. 2), although this correlation breaks down at higher temperatures. *V*_pmaxA_ and *A*_max_ were similar among the three C_4_ subtypes and two growth forms, partially rejecting Hypothesis 1 (Fig. 3a–d), but were influenced by growth and measurement conditions. Indoor plants exhibited higher *V*_pmaxA_ and *A*_max_ than their outdoor counterparts, supporting Hypothesis 2 (Fig. 3e,f). Both *V*_pmaxA_ and *A*_max_ decreased with increasing mean growth *T*_max_ (Fig. 5a,b), while they increased with increasing measurement *T*_leaf_ (Fig. 6a,b), providing partial support for Hypothesis 3. Interestingly, the response of *A*_max_ to *T*_leaf_ depended on mean *T*_max_, indicating that thermal acclimation alters the thermal sensitivity of photosynthetic capacity in C_4_ species (Fig. 7). There was no significant effect of growth CO_2_ concentration on photosynthetic capacity, leading us to accept Hypothesis 4, although more data are needed at low and high growth CO_2_ concentrations to further confirm this result (Fig. 5c,d). Lastly, we explored whether the more commonly measured C_4_ model species (maize, sorghum and *S. viridis*) were good representatives of a wide range of C_4_ plants. We found no evidence of generalised superior photosynthetic capacity in C_4_ model species when compared to non‐model species (e.g. native C_4_ grasses and eudicots as a whole; Table 1), although C_4_ model species had higher photosynthetic capacity than non‐model species when grown outdoors (Fig. 4). Overall, our analysis highlights that C_4_ photosynthesis is strongly affected by growth and measurement conditions but is largely similar across C_4_ species from different biochemical subtypes and growth forms. We discuss the potential reasons and consequences of our findings below.

### Coupling between *V*_pmaxA_ and *A*_max_ reflects photosynthetic limitations

We found a strong positive correlation between estimated *V*_pmaxA_ and *A*_max_ in the *T*_leaf_ range of 25–30°C (Fig. 2b). At *T*_leaf_ above 30°C, the positive correlation between *V*_pmaxA_ and *A*_max_ weakened, with *A*_max_ showing less pronounced change per unit increase in *V*_pmaxA_ (Fig. 2c,d). The C_4_ photosynthetic cycle (reflected as *V*_pmaxA_) is generally more efficient than the C_3_ cycle (reflected as *A*_max_) under high temperatures and light intensities, due to its CO_2_‐concentrating mechanism that suppresses photorespiration and maintains high RuBisCO efficiency (von Caemmerer & Furbank, 2003). When the efficiency of the C_4_ cycle exceeds that of the C_3_ cycle (i.e. the rate of CO_2_ delivery to bundle sheath cells outpaces the rate of RuBisCO carboxylation), CO_2_ may begin to leak back into mesophyll cells, increasing the energetic cost required to refix this CO_2_ into C_4_ acids in mesophyll cells. This imbalance between C_3_ and C_4_ cycles, particularly under low light (i.e. *PPFD* < 100 μmol photon m^−2^ s^−1^; Yin *et al*., 2016), can reduce overall photosynthetic efficiency (i.e. *A*_max_). Given that most of our *A*/*C*_i_ measurements were done at *PPFD* > 1500 μmol photon m^−2^ s^−1^ (Fig. S12), we argue that our observation is more related to different temperature sensitivities of *V*_pmaxA_ and *A*_max_ (to be described later).

Our result also agrees with findings derived from flux control analysis (von Caemmerer & Furbank, 2016) and data syntheses (Pignon & Long, 2020), and highlights differences in the temperature‐dependent responses of biochemical processes underpinning *V*_pmaxA_ and *A*_max_, such that *A*_max_ was less temperature sensitive at high *T*_leaf_ than *V*_pmaxA_. The *A*_max_ in C_4_ plants is primarily determined by the maximum carboxylation activity of RuBisCO (*V*_cmax_) and the rate of RuBP regeneration at high *C*_i_, with a reduction in either of these leading to a lower *A*_max_ (Furbank *et al*., 1996; von Caemmerer *et al*., 1997; von Caemmerer & Furbank, 1999). At high *T*_leaf_, RuBisCO inactivation due to a decrease in RuBisCO activase activity (Hendrickson *et al*., 2008; Salesse‐Smith *et al*., 2018) reduces *V*_cmax_ and ultimately *A*_max_. The RuBP regeneration rate can be limiting when measurement *PPFD* or the capacity of Calvin cycle enzymes, other than RuBisCO, limit net CO_2_ assimilation rate at high *C*_i_, or when the thylakoid membrane is damaged at high temperatures (Peixoto & Sage, 2017). The limitation imposed by RuBP regeneration is expected to be pronounced in C_4_ plants, which have lower concentrations of RuBP than C_3_ plants (Arrivault *et al*., 2019). However, this limitation is likely not related to the abundance and activity of sedoheptulose‐1,7‐bisphosphatase (a rate‐limiting enzyme in Calvin cycle; Ermakova *et al*., 2022). An RuBP limitation could further constrain photosynthesis as *C*_i_ increases, as an increase in *C*_i_ promotes RuBisCO carboxylase activity and leads to more RuBP being consumed. In our dataset, the majority of *A*/*C*_i_ measurements were done at *PPFD* > 1500 μmol photon m^−2^ s^−1^, including those measured at *T*_leaf_ > 35°C (Fig. S12). However, photosynthesis in C_4_ plants is usually not light‐saturated even at 1500 μmol photons m^−2^ s^−1^ (Ermakova *et al*., 2019, 2023). There is evidence that due to adaptation to tropical/subtropical climates where high‐light environments are common, C_4_ plants evolved a series of photosynthetic traits that allow them to grow under high light (Wasilewska‐Dębowska *et al*., 2022). Adaptation to high‐light conditions may arguably put C_4_ plants at a disadvantage in light conditions that are suboptimal for them but saturating for C_3_ plants. These findings highlight the complex interplay between temperature, biochemical limitations and light availability in regulating C₄ photosynthesis, which in part has become a bottleneck for C_4_ photosynthesis modelling at the ecosystem level (Knauer *et al*., 2023).

### Photosynthetic capacity is similar across biochemical types and growth forms

Our results show that variation in C_4_ biochemical subtypes and growth forms does not lead to systematic variation in photosynthetic capacity (Fig. 3a–d). In general, popular model species (C_4_ NADP‐ME type grasses: maize, sorghum and *S. viridis*) exhibit similar photosynthetic capacity when compared to non‐model C_4_ species representing the three C_4_ biochemical subtypes (Table 1). However, when growth location is considered, the photosynthetic capacity of C_4_ model species is higher than that of non‐model species when both are grown outdoors (Fig. 4). These results suggest that photosynthetic capacity data collected from model species could overestimate the productivity of non‐model species in outdoor conditions. These findings are particularly important for the modelling community, given that the current C_4_ vegetation model at the ecosystem level was developed based on parameters measured in maize grown in controlled environments (Collatz *et al*., 1992; Lawrence *et al*., 2019), and likely overestimates the productivity of C_4_ native and eudicots grown in the field. The lack of a significant effect of C_4_ subtypes on photosynthetic capacity further emphasises the biochemical flexibility in the C_4_ pathway. There is growing molecular, biochemical and physiological evidence, suggesting that the three classical subtypes, originally defined based on early ^14^C‐labelling studies (Hatch & Slack, 1970; Hatch, 1971), can be biochemically flexible (Furbank, 2011; Bräutigam *et al*., 2014; Wang *et al*., 2014; Sales *et al*., 2021), which in turn diminishes any potential differences in photosynthetic capacity among the subtypes. This biochemical flexibility may be regulated by developmental and environmental variation, further highlighting the role of environmental effects on C_4_ photosynthesis (to be described later).

### Growth location affects photosynthetic capacity

Our results showed that growth and measurement conditions are the major factors affecting photosynthetic capacity in C_4_ plants. Indoor plants exhibit consistently higher *V*_pmaxA_ and *A*_max_ than their outdoor counterparts (Fig. 3e,f), despite indoor plants being grown in warmer environments, which might suppress photosynthetic capacity (Fig. S4a). Plants grown in indoor, controlled environments are usually well‐watered and fertilised, and are not challenged with pests and extreme conditions (e.g. heat waves or frost stress; Poorter *et al*., 2016). The lower photosynthetic capacity in outdoor non‐model species is likely due to the more stressful conditions they experience, which are primarily characterised by lower water and nutrient availability than indoor‐grown plants. For example, long‐term drought (i.e. > 100 d) could cause significant reductions in stomatal conductance, net photosynthetic rate and *V*_pmaxA_, with such effects being exacerbated in N‐limited C_4_ plants (Markelz *et al*., 2011). Interestingly, the impact of drought on photosynthetic capacity (i.e. *A*_max_) was not seen in two well‐fertilised C_4_ grasses, *Dactyloctenium aegyptium* and *Schoenefeldia gracilis* (Maroco *et al*., 2000), suggesting changes in photosynthetic capacity may be associated with interactive effects of drought and N limitation on plants. By contrast, C_4_ model species, particularly maize and sorghum, have been selectively bred for improved drought tolerance (Lopes *et al*., 2011) and are typically well‐watered and well‐fertilised in crop fields due to their high agricultural value. These effects may have contributed to higher photosynthetic capacity in C_4_ model species grown outdoors (Fig. 4). Further research is needed to dissect the mechanism underpinning the combined effects of drought and N limitation on photosynthesis and to identify why outdoor growth conditions affect non‐model species more than model C_4_ species.

### Temperature significantly influences photosynthetic capacity

We observed that both *V*_pmaxA_ and *A*_max_ decreased with increasing mean *T*_max_ (Fig. 5a,b). This finding is supported by previous work (Berry & Bjorkman, 1980) and aligns with our knowledge of thermal acclimation (Way & Yamori, 2014). Plants grown in warmer conditions often reduce photosynthetic capacity (i.e. *V*_pmaxA_ and *A*_max_), while maintaining comparable (or even higher) *A* at their growth temperatures compared with control plants. This decrease in photosynthetic capacity is likely due to reduced photosynthetic enzyme concentrations. Warmer temperatures allow plants to achieve the same rate of photosynthesis with lower enzyme concentrations because higher temperatures enhance enzyme activity (Way & Yamori, 2014; Yamori *et al*., 2014). Evidence supports this idea in C_4_ plants: Dwyer *et al*. (2007) compared C_4_ *Panicum coloratum*, *Cenchrus ciliaris* and *Flaveria bidentis* grown at moderate and high temperatures and found that warm‐grown plants had reduced photosynthetic capacity (i.e. *A*_max_), underpinned by lower concentrations of RuBisCO and chloroplastic electron transport chain proteins. However, the authors did not find a significant effect of growth temperature on the activity or concentration of PEPc (measured at the respective growth temperatures), suggesting that PEPc may have high thermal stability (Chen *et al*., 1994; Chinthapalli *et al*., 2002; Boyd *et al*., 2015). Thus, it is possible that the decrease in *V*_pmaxA_ with increasing mean *T*_max_ observed in our study may be reflective of changes in PEP regeneration via PPDK rather than PEPc capacity *per se*. In *Miscanthus* × *giganteus*, the activity and capacity of PPDK per unit leaf area increases in plants grown at chilling temperature, compared with their warm‐grown controls (Wang *et al*., 2008). Furthermore, whether *A*/*C*_i_ measurements are taken at saturated *PPFD* may also affect PEP regeneration, which requires two photosynthetically generated ATP per PEP converted (Hatch, 1987). Although some of the measurements in our dataset were made at *PPFD* < 1000 μmol m^−2^ s^−1^, we found no significant effect of lower measurement *PPFD* on the responses of *V*_pmaxA_ and *A*_max_ to intrinsic or extrinsic factors (Tables 1, S3). Further research is needed to explore how PEPc and PPDK capacities are coordinated under thermal acclimation.

Our results highlight that both *V*_pmaxA_ and *A*_max_ increase with measurement *T*_leaf_ (Fig. 6a,b), which is indicative of enhanced enzymatic activities at higher measurement temperatures. According to an *in vitro* study in *S. viridis*, the capacities of major C_4_ photosynthetic enzymes generally increase with rising *T*_leaf_ due to a corresponding rise in enzyme activities (Boyd *et al*., 2015). The carboxylation activities of RuBisCO and PEPc increase exponentially between 10°C and 40–45°C, before declining (Chen *et al*., 1994; Chinthapalli *et al*., 2002; Boyd *et al*., 2015). However, the extent to which this temperature response of enzymes holds true *in vivo* remains uncertain and needs to be confirmed with high‐resolution photosynthesis‐temperature response curves across a diverse range of C_4_ species.

Finally, our analysis highlights that the response of *A*_max_ to *T*_leaf_ is influenced by mean *T*_max_, whereas that of *V*_pmaxA_ is not (Table 1; Fig. 7). We found that *A*_max_ increases to a greater extent with increasing *T*_leaf_ in cool‐grown plants than the plants grown in warmer conditions, suggesting that *A*_max_ of cool‐grown plants is more sensitive to changes in *T*_leaf_ (Fig. 7b). Our data suggest that *A*_max_ of C_4_ plants grown under future, hotter climates will be less affected by daily temperature fluctuations compared with plants grown under the current climate (i.e. *A*_max_ will be more stable; see circles in Fig. 7). This reduced thermal sensitivity of *A*_max_ at higher temperatures may help buffer photosynthesis in future climates, where heat waves are predicted to occur more frequently and intensely (Brown, 2020). However, this response could also limit the ability of warm‐grown C_4_ plants to achieve high *A*_max_ at the high *T*_leaf_ conditions they will experience (Fig. 7), which may suppress CO_2_ uptake in future climate conditions. As we saw no effect of elevated growth CO_2_ concentrations on either *V*_pmaxA_ or *A*_max_ (Fig. 5c,d), in line with field studies of C_4_ species (Leakey *et al*., 2006; Leakey, 2009; Markelz *et al*., 2011), these temperature effects are likely to be stronger controls on C_4_ photosynthetic capacity in future climates than predicted increases in CO_2_ levels. However, further research is needed to fully understand how climate change affects the photosynthetic physiology of C_4_ plants.

### Future perspectives

Looking forward, our analyses highlight areas where more studies are warranted. For example, the physiology of C_4_ eudicots and sedges is underrepresented in our dataset, although eudicots and sedges comprise 38% of C_4_ plants (Sage, 2017) and thrive in extreme environments (e.g. *Haloxylon*, a C_4_ desert shrub (Feng *et al*., 2023)). Understanding how C_4_ photosynthesis responds to these extreme environments could provide insights into improving how other plants (e.g. C_3_ crops) cope with extreme environments. Furthermore, our results show that *A*_max_ responds differently to growth and measurement temperatures than does *V*_pmaxA_. We explored potential biochemical limitations that underpin *A*_max_ and *V*_pmaxA_ separately, in addition to the response of these limitations to changes in temperature. However, it is unclear to what extent the individual biochemical limitations may interact and affect overall photosynthetic capacity. If one assumes that PEP regeneration is not limited, *V*_pmaxA_ is largely determined by the property of PEPc (e.g. activation state and activity of the enzyme) and its temperature sensitivity likely reflects the temperature sensitivity of PEPc. By contrast, *A*_max_ is determined by a variety of biochemical processes (e.g. RuBisCO carboxylation and rates of electron transport). Therefore, understanding how these processes interact in response to changing temperatures is crucial for fully grasping the overall response of *A*_max_.

### Conclusion

C_4_ plants play a crucial role in carbon exchange and food security on a global scale. A growing number of studies dissect the mechanism of C_4_ photosynthesis in specific contexts, yet how C_4_ photosynthetic capacity responds to differences in biochemical subtypes, plant functional types, and growth and measurement conditions among a wide range of species remains unclear. Our study uncovers broad patterns of photosynthetic capacity from 74 C_4_ species and highlights that environmental conditions play a dominant role in determining C_4_ photosynthetic capacity. Importantly, we demonstrate that while the current simplified parameterisation of C_4_ NADP‐ME‐type photosynthesis in leaf and ecosystem‐level models likely represents species of all three biochemical types, it overestimates the photosynthetic capacity of C_4_ native species in field conditions. Future research should aim to refine current parameter values by incorporating detailed equations that capture the effects of abiotic factors, such as water and nutrient availability, on photosynthetic capacity (Smith *et al*., 2019), while also examining their link to the leaf economics spectrum (Monson *et al*., 2025). Additionally, we compile a C_4_ *A*/*C*_i_ database for community use, addressing the shortage of accessible raw data for C_4_ gas exchange. For example, the TRY leaf‐trait database (http://www.try‐db.org), one of the most comprehensive databases of plant physiological trait data in the world, contains leaf photosynthetic point measurements for only 591 C_4_ species, out of 69,000 terrestrial species measured, and no C_4_ photosynthetic response curves (Kattge *et al*., 2011, 2020). Our database opens new avenues for future studies involving big data analysis for C_4_ plants, such as simulation modelling.

## Competing interests

None declared.

## Author contributions

DAW conceived the concept for the paper within the discussions provided by the US Geological Survey Powell Center C_4_ Photosynthesis Working Group. YF compiled the dataset, coded the scripts and ran the analyses with help from DWAN and BEM. NGS, EAA, FAB, FRD, ME, PF, RTF, SHG, OG, LG, VJ, ADBL, SL, ML, VSP, MMP, BVS, SC, RW and DAW provided the data to this study. YF and DAW wrote the first draft, and received feedback from RKM, RFS, DMG, JK, DLL, KP and CJS. All authors contributed substantially to improving the draft.

## Disclaimer

The New Phytologist Foundation remains neutral with regard to jurisdictional claims in maps and in any institutional affiliations.

## Supporting information


**Dataset S1** C_4_ *A*/*C*_i_ data collated for this study.
**Dataset S2** Estimated apparent *V*_pmaxA_ and *A*_max_ used in the analysis.

**Fig. S1** Species distribution within the categories of growth forms and growth locations.
**Fig. S2** Correlation of mean *T*_max_ and *T*_min_.
**Fig. S3** Data distribution of *V*_pmaxA_ and *A*_max_ in categories of C_4_ subtypes and growth forms over *T*_leaf_, mean *T*_max_ and *PPFD*.
**Fig. S4** Histograms describing the data distribution across the spectrum of mean *T*_max_ and *T*_leaf_ for growth location, growth form and important species group.
**Fig. S5** *V*_pmaxA_ and *A*_max_ plotted by C_4_ biochemical subtypes, with symbols highlighting the effect of phylogeny.
**Fig. S6** Comparison between *V*_pmaxA_ and *A*_max_ with growth location coloured by species group (maize vs sorghum vs other species).
**Fig. S7** Relationships between *V*_pmaxA_ and *A*_max_ with mean *T*_max_ for measurements done at *T*_*l*eaf_ between 25°C and 30°C.
**Fig. S8** Relationships between *V*_pmaxA_ and *A*_max_ with mean *T*_max_ for indoor and outdoor plants.
**Fig. S9** Relationships between *V*_pmaxA_ and *A*_max_ with growth CO_2_ levels for indoor and outdoor plants.
**Fig. S10** Relationships between *V*_pmaxA_ and *A*_max_ with *T*_leaf_ for indoor and outdoor plants.
**Fig. S11** Relationships between *V*_pmaxA_ and *A*_max_ with *PPFD* for indoor and outdoor plants.
**Fig. S12** Correlation of mean *T*_max_ and *T*_min_ for data with *PPFD* > 1500 μmol photon m^−2^ s^−1^.
**Table S1** Results of multivariate linear mixed‐effects models testing the response of *V*_pmaxA_ and *A*_max_ against species‐specific traits and experimental conditions for indoor and outdoor plants separately.
**Table S2** Correlations between *V*_pmaxA_ or *A*_max_ and species traits in indoor and outdoor plants.
**Table S3** Results of multivariate linear mixed‐effects models for data with *PPFD* > 1000 μmol photon m^−2^ s^−1^.
Please note: Wiley is not responsible for the content or functionality of any Supporting Information supplied by the authors. Any queries (other than missing material) should be directed to the *New Phytologist* Central Office.

## Data Availability

The data synthesised in this work are openly available as Supplementary Datasets and can be downloaded from https://github.com/yuzhenfanANU/C4_repository.
