# Supplementary material for: Environmental factors have a greater influence on photosynthetic capacity in C4 plants than biochemical subtypes or growth forms
Source: New Phytol. 2025 Aug 31;248(3):1205–24. doi: 10.1111/nph.70525 (PMC12489297; doi:10.1111/nph.70525)
Supplement: Supplementary file 2 — Fig. S1 Species distribution within the categories of growth forms and growth locations. Fig. S2 Correlation of mean T max and T min. Fig. S3 Data distribution of V pmaxA and A max in categories of C4 subtypes and growth forms over T leaf, mean T max and PPFD. Fig. S4 Histograms describing the data distribution across the spectrum of mean T max and T leaf for growth location, growth form and important species group. Fig. S5 V pmaxA and A max plotted by C4 biochemical subtypes, with symbols highlighting the effect of phylogeny. Fig. S6 Comparison between V pmaxA and A max with growth location coloured by species group (maize vs sorghum vs other species). Fig. S7 Relationships between V pmaxA and A max with mean T max for measurements done at T leaf between 25°C and 30°C. Fig. S8 Relationships between V pmaxA and A max with mean T max for indoor and outdoor plants. Fig. S9 Relationships between V pmaxA and A max with growth CO2 levels for indoor and outdoor plants. Fig. S10 Relationships between V pmaxA and A max with T leaf for indoor and outdoor plants. Fig. S11 Relationships between V pmaxA and A max with PPFD for indoor and outdoor plants. Fig. S12 Correlation of mean T max and T min for data with PPFD > 1500 μmol photon m−2 s−1. Table S1 Results of multivariate linear mixed‐effects models testing the response of V pmaxA and A max against species‐specific traits and experimental conditions for indoor and outdoor plants separately. Table S2 Correlations between V pmaxA or A max and species traits in indoor and outdoor plants. Table S3 Results of multivariate linear mixed‐effects models for data with PPFD > 1000 μmol photon m−2 s−1. Please note: Wiley is not responsible for the content or functionality of any Supporting Information supplied by the authors. Any queries (other than missing material) should be directed to the New Phytologist Central Office. [file NPH-248-1205-s002.pdf]

***New Phytologist* Supporting Information**

Article title: Environmental factors have a greater influence on photosynthetic capacity in C_4 plants than biochemical subtypes or growth forms

Authors: Yuzhen Fan, Daniel W. A. Noble, Belinda E. Medlyn, Russell K. Monson, Rowan F. Sage, Nicholas G. Smith, Elizabeth A. Ainsworth, Florian A. Busch, Florence R. Danila, Maria Ermakova, Patrick Friesen, Robert T. Furbank, Shu Han Gan, Oula Ghannoum, Daniel M. Griffith, Lianhong Gu, Vinod Jacob, Jürgen Knauer, Andrew D. B. Leakey, Shuai Li, Danica L. Lombardozzi, Martha Ludwig, Varsha S. Pathare, Murilo M. Peixoto, Karine Prado, Balasaheb V. Sonawane, Christopher J. Still, Susanne von Caemmerer, Russell Woodford, Danielle A. Way

Article acceptance date: 8 August 2025

The following Supporting Information is available for this article:

Dataset S1. C_4 A/C_i data collated for this study.

Dataset S2. Estimated V_{pmaxA} and A_{max} used in the analysis.

Figure S1. Species distribution within the categories of (a) growth forms and (b) growth locations. The top three measured species (maize, sorghum and *Setaria viridis*) are labelled explicitly, whereas all other species are shown as "Others". Detailed information on species is available in Supplementary Datasets S1 and S2.

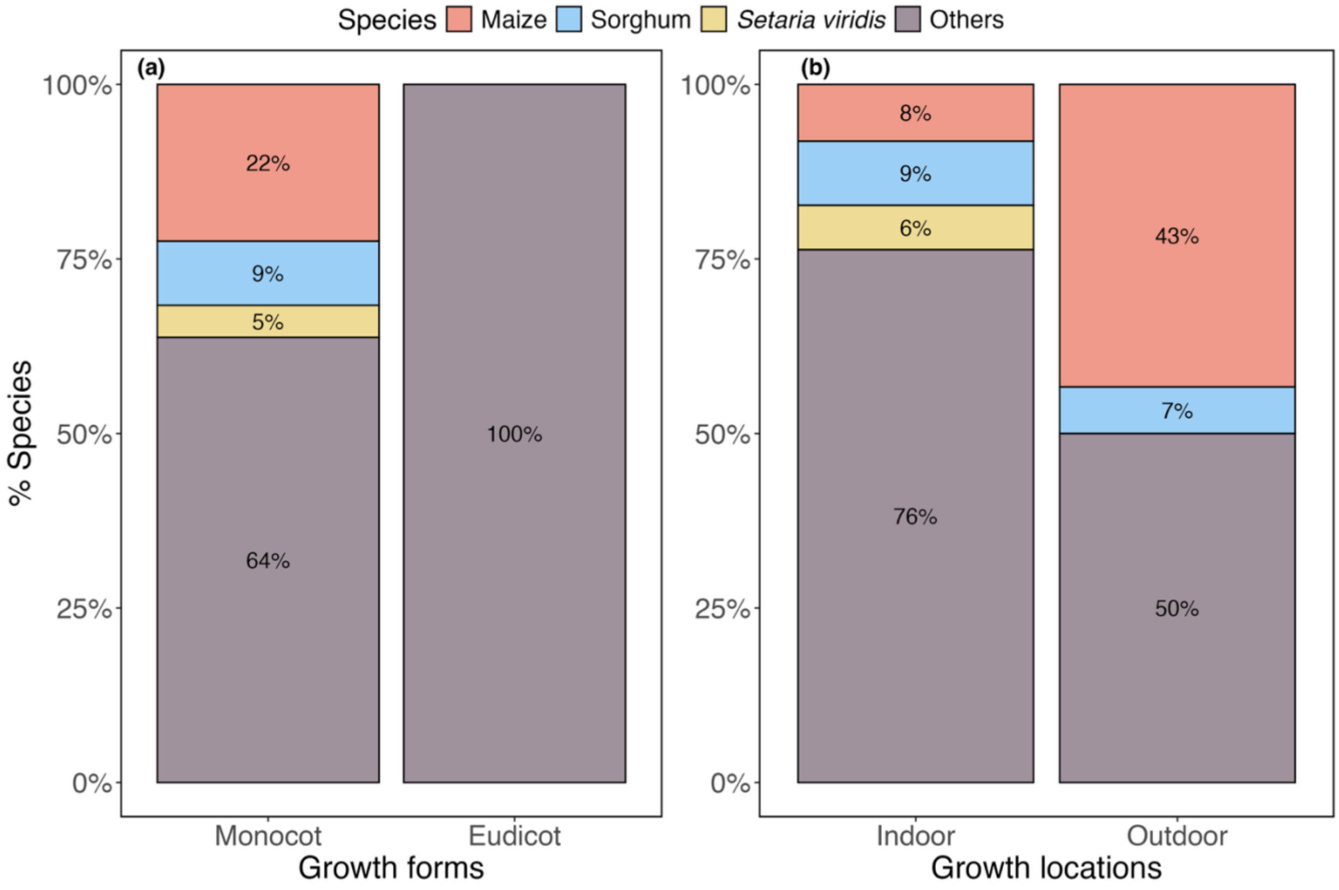

Figure S2. Correlation of mean T_{max} and T_{min} (°C). The size of the dots reflects the number of A/C_i curve groups. See Supplementary Dataset S1 for more details.

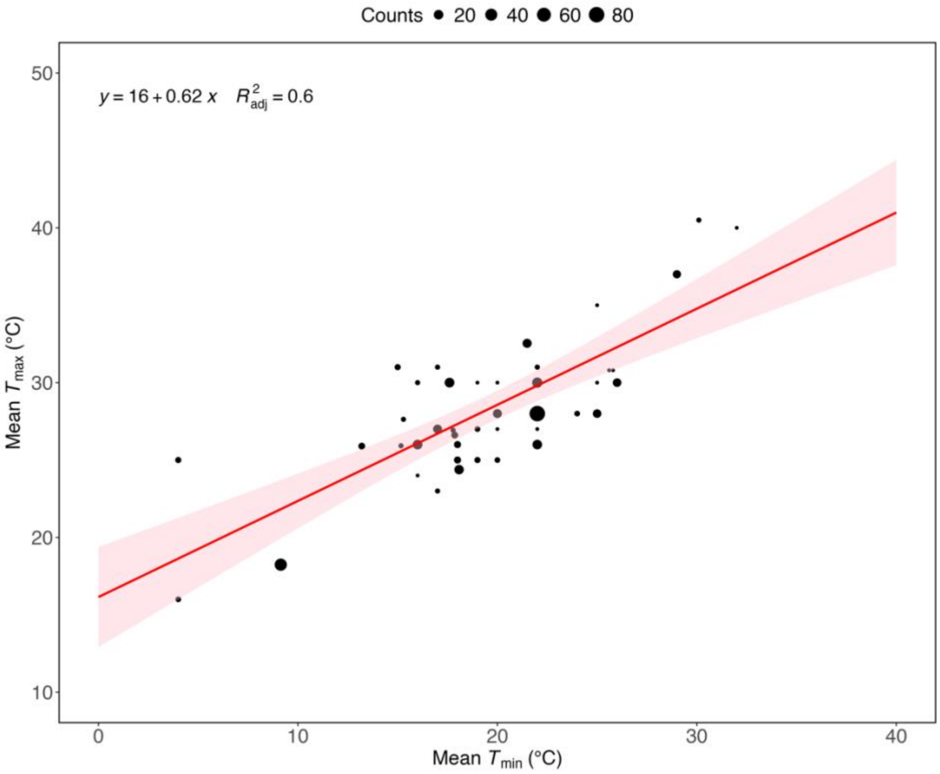

Figure S3. Data distribution of V_{pmaxA} and A_{max} in categories of C_4 subtypes and growth forms over T_{leaf}, mean T_{max} and *PPFD*. Linear regression is fitted to each category within each panel, with equations, adjusted R^2 and P values being shown.

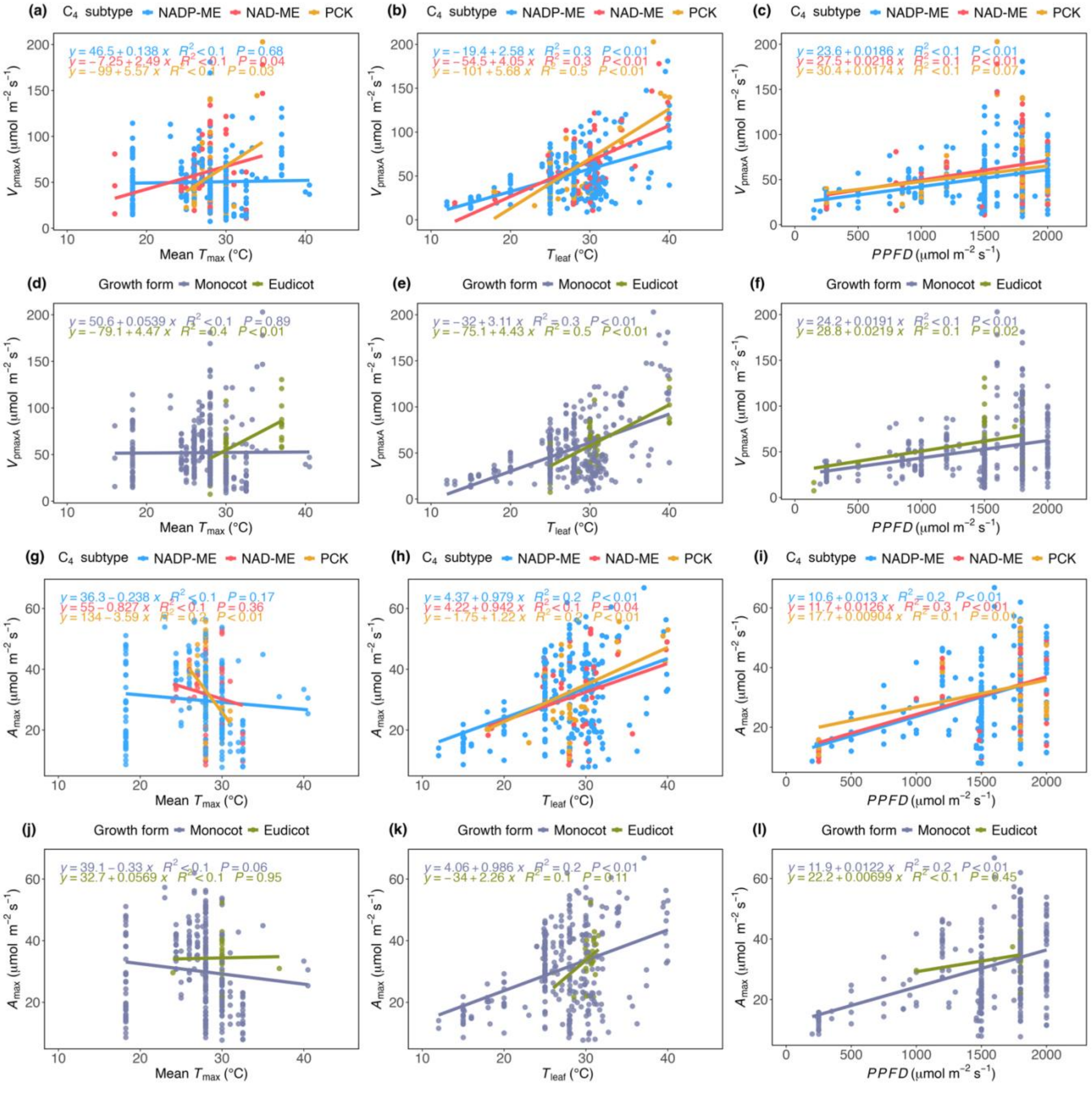

Figure S4. Histograms describing the data distribution across the spectrum of mean T_{max} and T_{leaf} for growth location (a and b, respectively), growth form (c and d, respectively) and important species group (e and f, respectively). Dash lines indicate the average values of mean T_{max} and T_{leaf} across plants grouped by different traits.

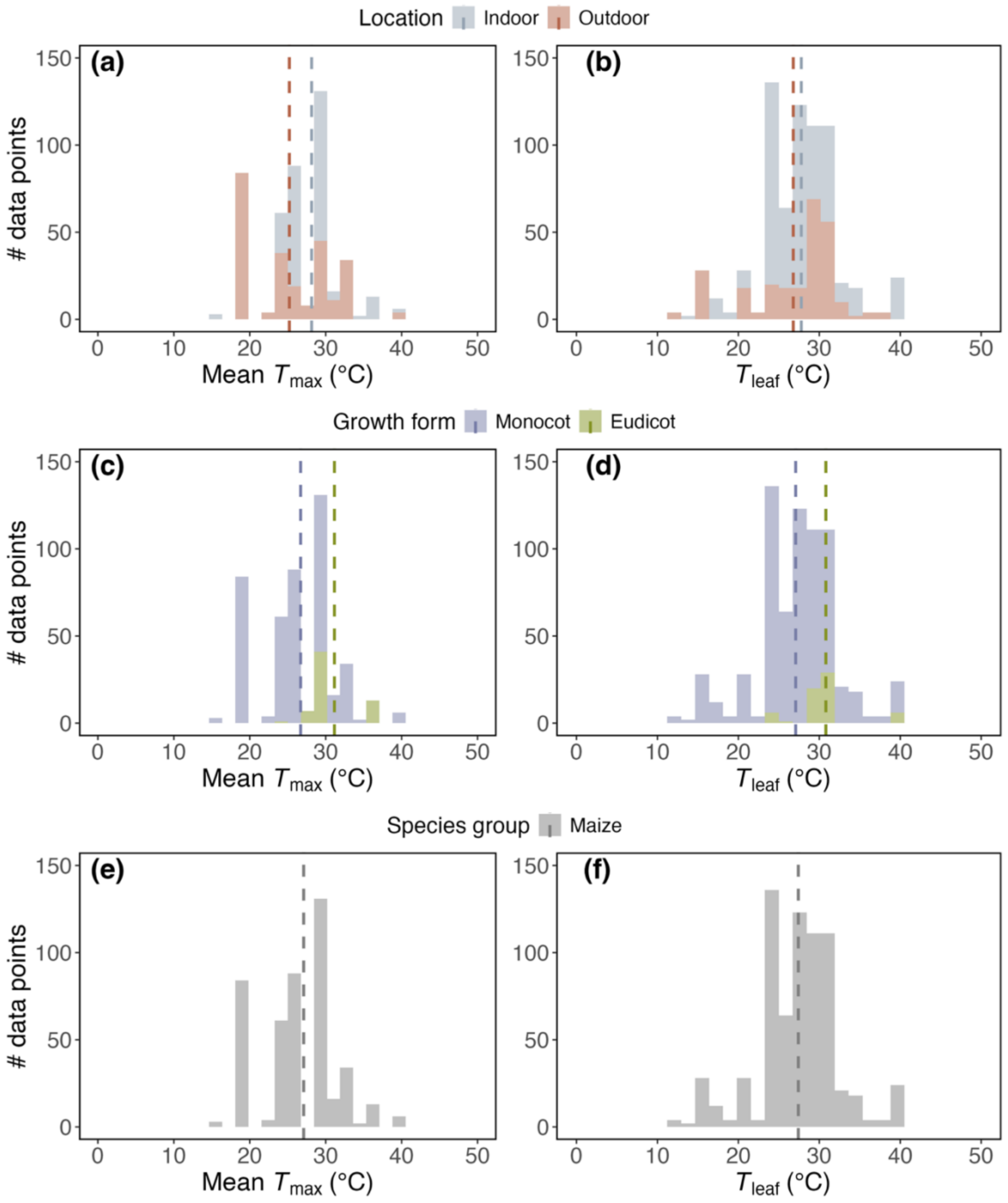

Figure S5. V_{pmaxA} and A_{max} plotted by C_4 biochemical subtypes (a and b, respectively). Data presented here are a mixture of measurements done at various growth and measuring temperatures and irradiance. Raw data points are plotted as coloured symbols, with different symbol shapes reflecting lineages. Model-predicted marginal means and intervals are shown as a horizontal line. On this line, black circles indicate model-predicted meta-analytic means of V_{pmaxA} or A_{max}, thick bars are 95% confidence intervals and thin bars are 95% prediction intervals. On the right-hand side of each panel, the number of unique species per category (n.spp) and the number of individual studies (e.size) are indicated. *P* values of a multivariate mixed effect model indicating the effect of lineages on V_{pmaxA} and A_{max} are shown on each panel.

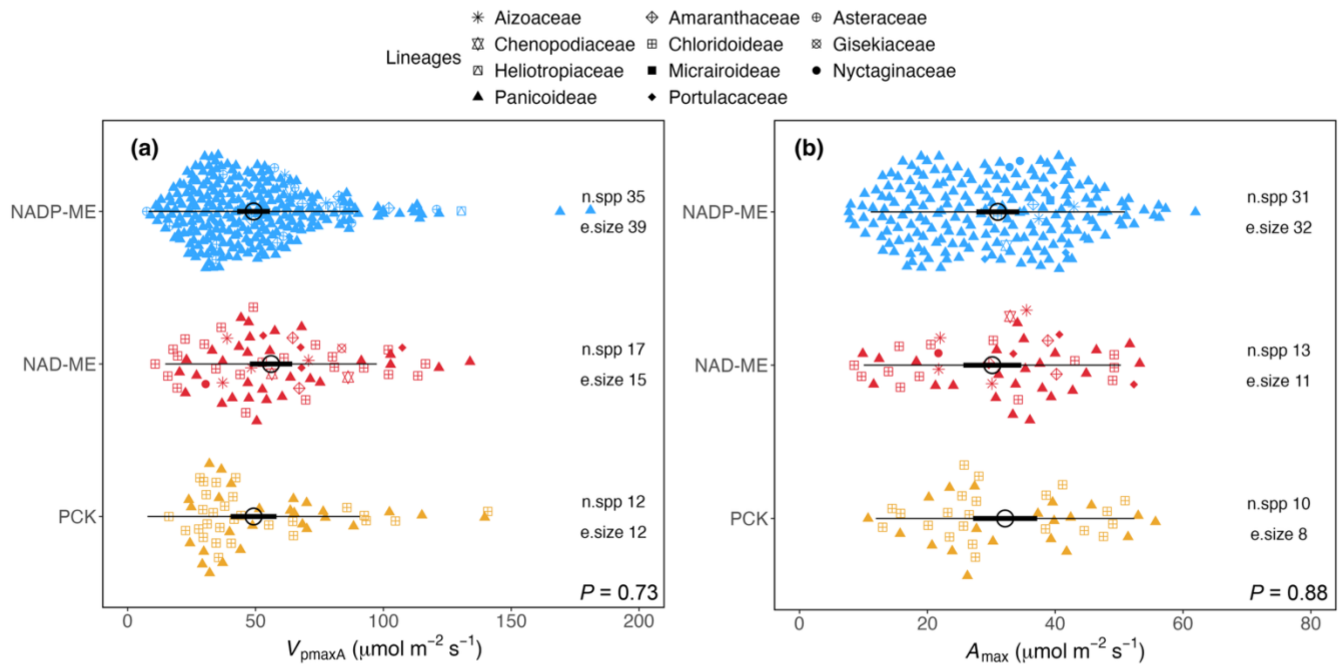

Figure S6. Comparison between V_{pmaxA} and A_{max} with growth location in maize, sorghum and other species. A linear mixed-effect model was conducted to examine V_{pmaxA} and A_{max} among the three species groups at a location, or within a species group at both locations. Statistical results of comparisons are denoted with horizontal lines (i.e. the two bars at the beginning and the end of a horizontal line are compared) and asterisks indicate statistical significance (*, $P < 0.05$; **, $P < 0.01$; ***, $P < 0.001$). The sample size (N) for each group is indicated on plots.

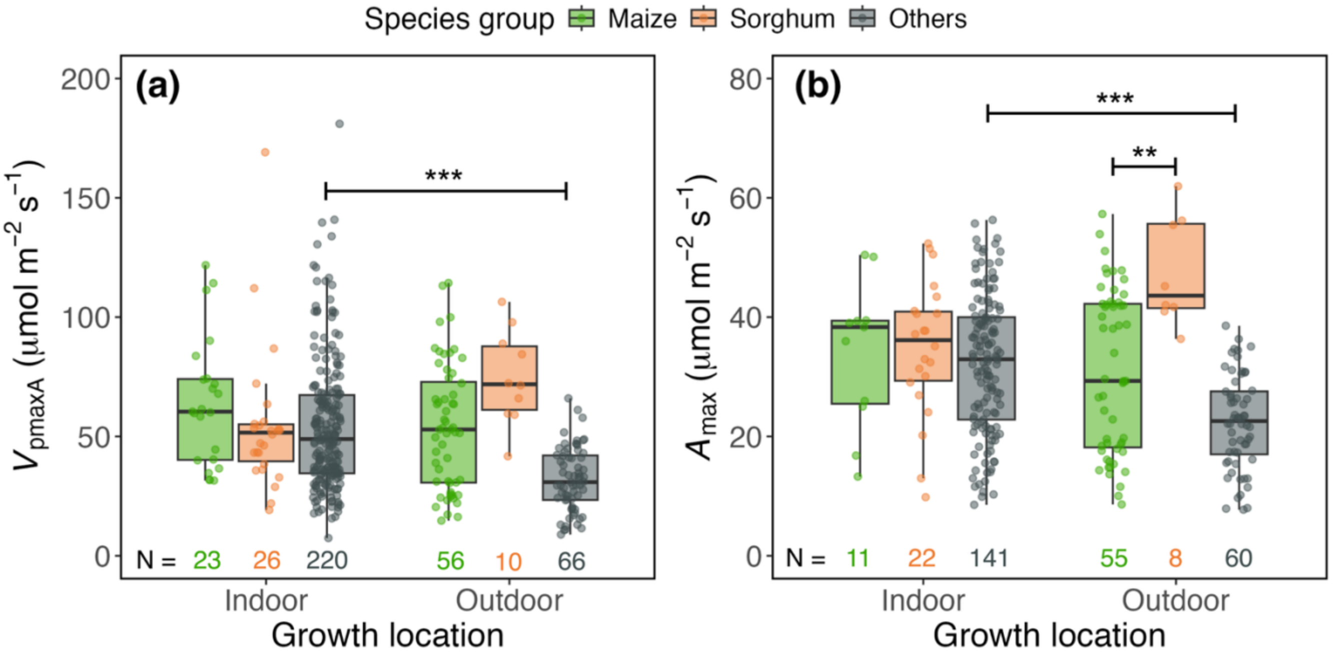

Figure S7. Relationships between V_{pmaxA} and A_{max} with mean T_{max} (a and b, respectively) for measurements done at T_{leaf} between 25 and 30°C. Data presented here are a mixture of measurements done at various *PPFD*. Data points are coloured in a gradient by T_{leaf}. Solid lines represent model-predicted values of V_{pmaxA} or A_{max} at a given T_{leaf} or *PPFD* (equations are shown at the top of each panel), dashed lines indicate 95% confidence intervals, and dotted lines show 95% prediction intervals. *P* values of the multivariate linear mixed-effects models are indicated. Significant codes: *, $P < 0.05$; **, $P < 0.01$; ***, $P < 0.001$.

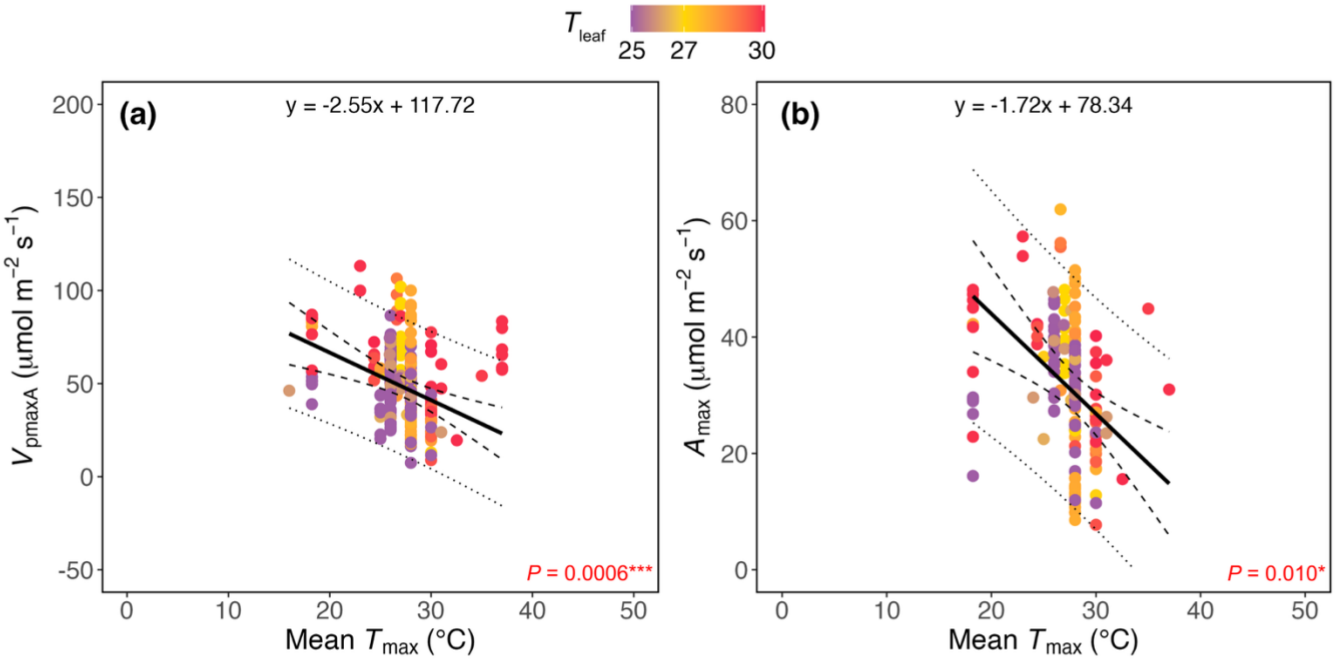

Figure S8. Relationships between V_{pmaxA} and A_{max} with mean T_{max} for indoor (a and b, respectively) and outdoor plants (c and d, respectively). Data points are coloured in a gradient by the measurement temperature (T_{leaf}). P values of the multivariate linear mixed-effects models are indicated (see Table S1). When there is a significant regression fit, solid lines represent model-predicted values of V_{pmaxA} or A_{max} at a given mean T_{max} (equations are shown at the top of each panel), dashed lines indicate 95% confidence intervals, and dotted lines show 95% prediction intervals. Significant codes: *, $P < 0.05$; **, $P < 0.01$; ***, $P < 0.001$.

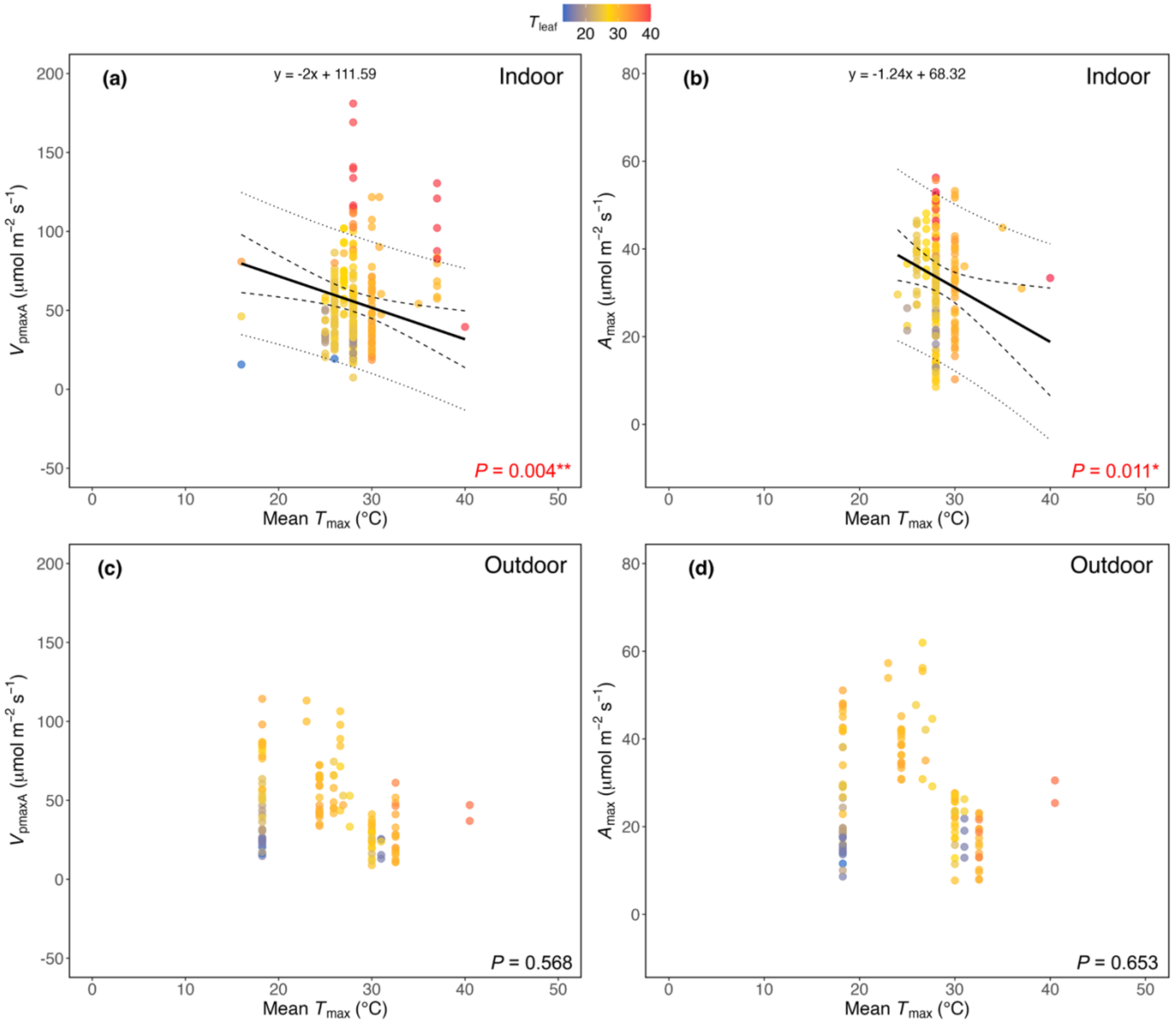

Figure S9. Relationships between V_{pmaxA} and A_{max} with growth CO_2 levels for indoor (a and b, respectively) and outdoor plants (c and d, respectively). *P* values of the multivariate linear mixed-effects models are indicated (see Table S1).

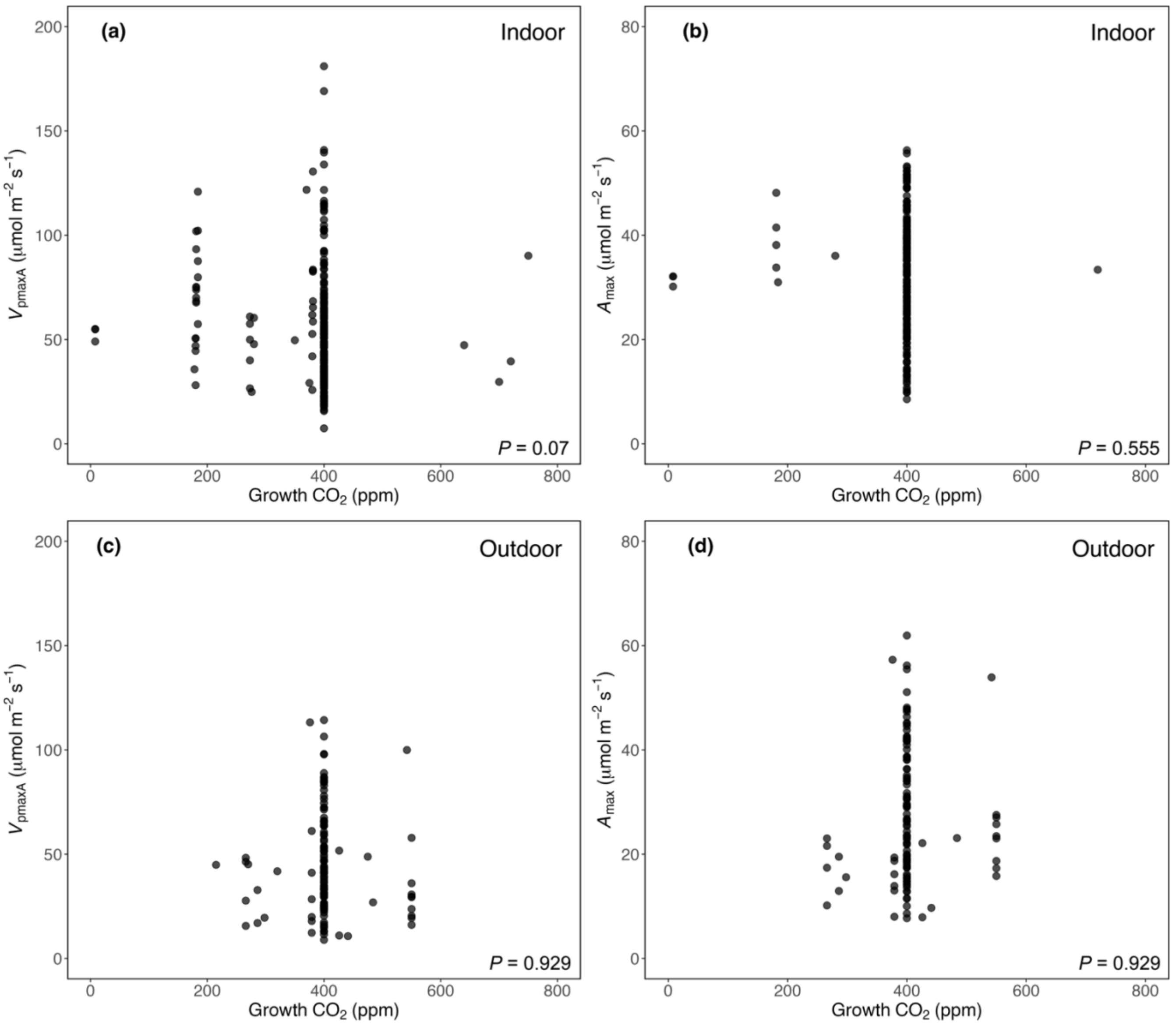

Figure S10. Relationships between V_{pmaxA} and A_{max} with T_{leaf} for indoor (a and b, respectively) and outdoor plants (c and d, respectively). Data points are coloured in a gradient by mean maximum growth temperature (mean T_{max}). P values of the multivariate linear mixed-effects models are indicated (see Table S1). When there is a significant regression fit, solid lines represent model-predicted values of V_{pmaxA} or A_{max} at a given mean T_{leaf} (equations are shown at the top of each panel), dashed lines indicate 95% confidence intervals, and dotted lines show 95% prediction intervals. Significant codes: *, $P < 0.05$; **, $P < 0.01$; ***, $P < 0.001$.

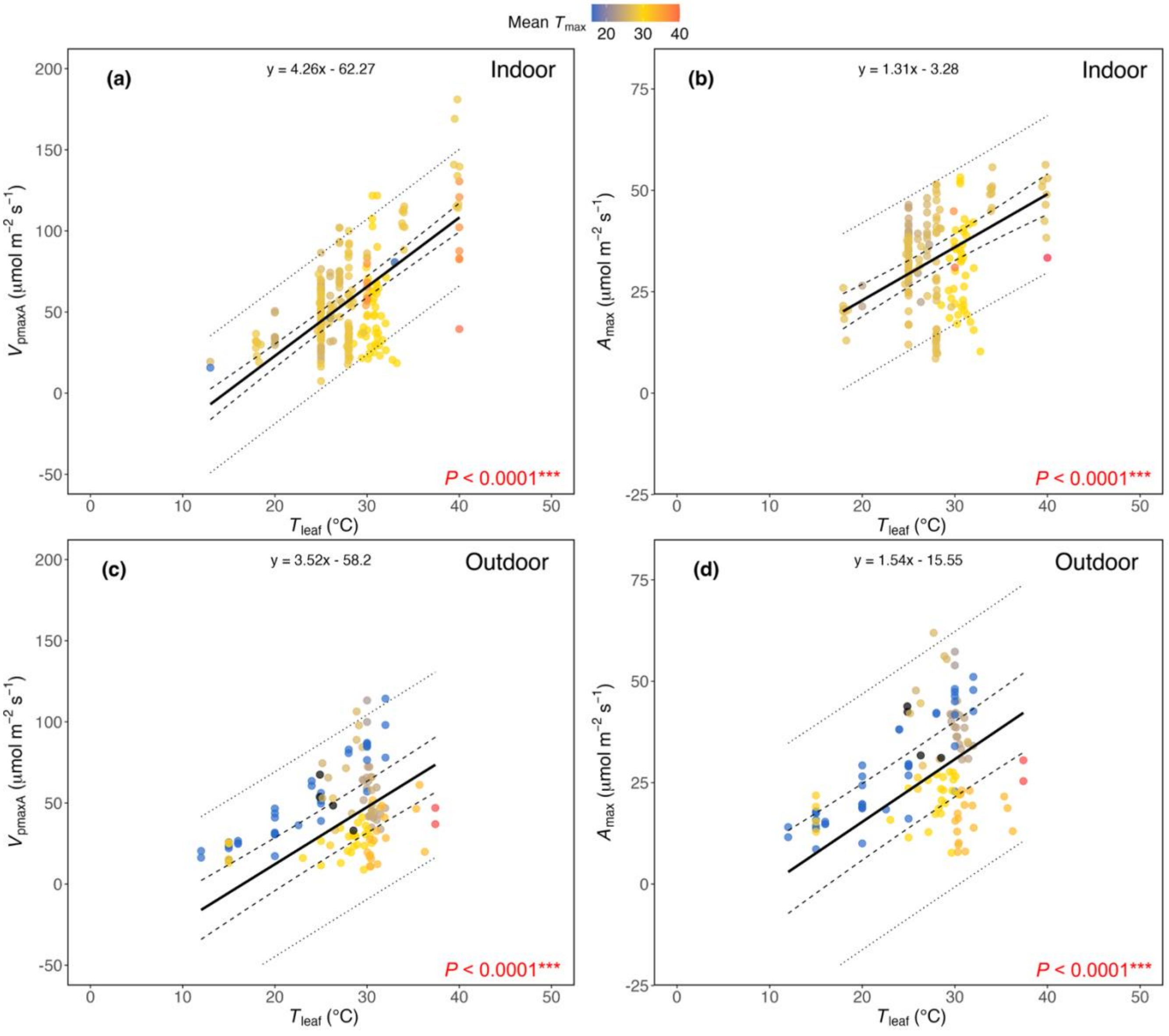

Figure S11. Relationships between V_{pmaxA} and A_{max} with *PPFD* for indoor (a and b, respectively) and outdoor plants (c and d, respectively). *P* values of the multivariate linear mixed-effects models are indicated (see Table S1). When there is a significant regression fit, solid lines represent model-predicted values of V_{pmaxA} or A_{max} at a given *PPFD* (equations are shown at the top of each panel), dashed lines indicate 95% confidence intervals, and dotted lines show 95% prediction intervals. Significant codes: *, $P < 0.05$; **, $P < 0.01$; ***, $P < 0.001$.

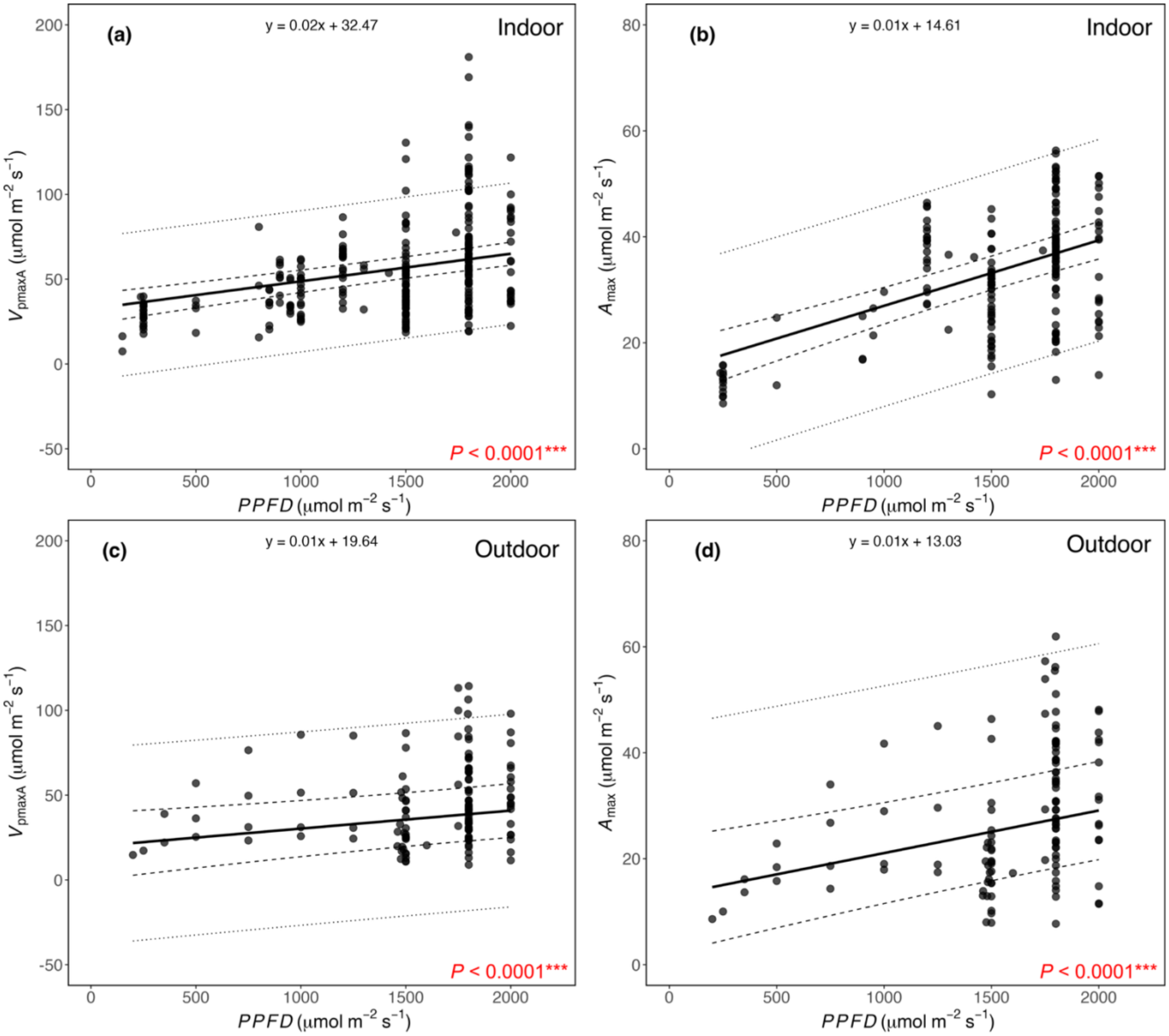

Figure S12. Correlation of A_{max} versus V_{pmaxA} estimated from data with *PPFD* > 1,500 μmol photon m^{-2} s^{-1}, coloured by T_{leaf}.

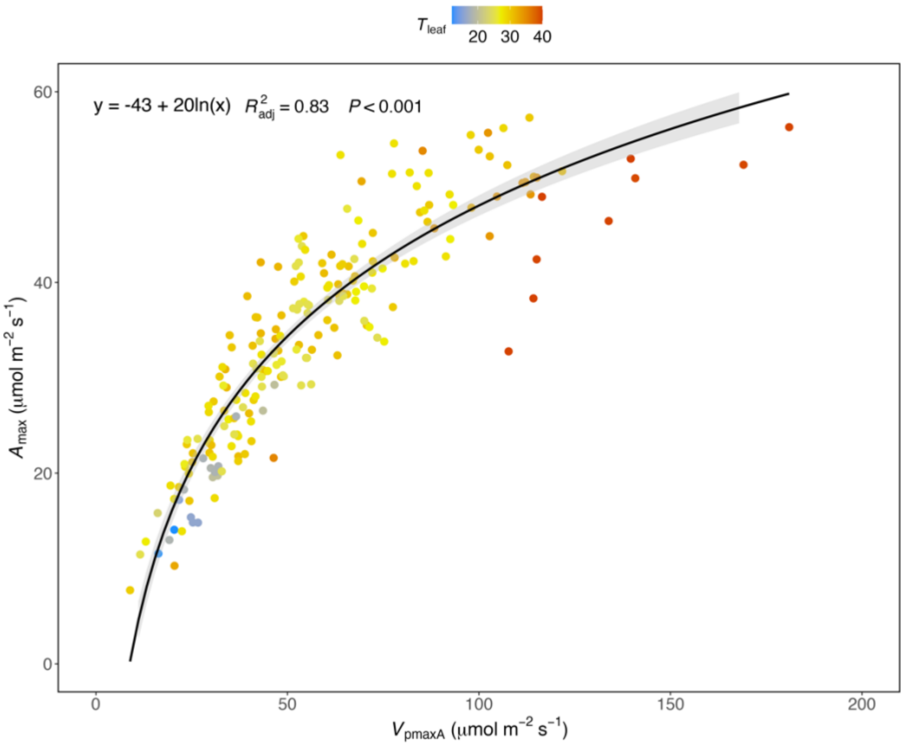

Table S1. Results of multivariate linear mixed-effects models testing the response of V_{pmaxA} and A_{max} against species-specific traits and experimental conditions for **indoor and outdoor plants separately**, with P values of the fixed effects shown. Continuous variables (i.e. mean T_{max}, growth CO_2, T_{leaf} and *PPFD*) were mean-centred before the analysis. Effect size denotes the number of studies, while the sample size is the number of V_{pmaxA} or A_{max} data points. Bold text indicates statistical significance. Significant codes: *, $P < 0.05$; **, $P < 0.01$; ***, $P < 0.001$. Note that only monocots (i.e. a single growth form) are present among outdoor plants, thus comparisons of growth form effects on V_{pmaxA} and A_{max} are not possible for outdoor-grown plants (denoted as 'NA'). In addition, assessment of interactions between fixed effect terms was not possible due to limited statistical power and to avoid model overfitting, given the unbalanced data points across categories and environments.

	**Indoor**		**Outdoor**	
	V_{pmaxA}	A_{max}	V_{pmaxA}	A_{max}
Fixed effects (P values)				
C_4 subtype	0.121	0.876	0.693	0.693
Growth form	0.220	0.284	NA	NA
Mean T_{max}	**0.004** **	**0.011** *	0.568	0.653
Growth CO_2	0.071	0.555	0.929	0.929
T_{leaf}	**< 0.0001*** **	**< 0.0001*** **	**< 0.0001*** **	**< 0.0001*** **
PPFD	**< 0.0001*** **	**< 0.0001*** **	**0.0003*** **	**< 0.0001*** **
Overall model statistics				
Effect size/sample size	32/269	21/174	17/132	20/123

Table S2. Correlations between V_{pmaxA} or A_{max} and species traits in indoor and outdoor plants. Slopes and intercepts were estimated using multivariate linear mixed-effects models (Table S3). Differences in slopes between indoor and outdoor plants for each trait group were assessed via trait × location interaction terms in the model presented in Table S2. Bold indicates statistical significance. Significance codes: *, $P < 0.05$; **, $P < 0.01$; *, $P < 0.001$.

Traits	Location	Sample size	Slope	Intercept	Slope homogeneity (*P* values)	Corresponding figures
$\boldsymbol{V_{pmaxA}}$						
Mean T_{max}	Indoor	269	-2	111.59	0.6214	Fig. S8 (a) vs (c)
	Outdoor	128	-0.47	48.39		
Growth CO_2	Indoor	269	-0.03	65.25	0.3820	Fig. S9 (a) vs (c)
	Outdoor	128	0	37.31		
T_{leaf}	Indoor	269	4.26	-62.27	**0.003****	Fig. S10 (a) vs (c)
	Outdoor	128	3.52	-58.2		
PPFD	Indoor	269	0.02	32.47	0.1131	Fig. S11 (a) vs (c)
	Outdoor	128	0.01	19.64		
$\boldsymbol{A_{max}}$						
Mean T_{max}	Indoor	174	-1.24	68.32	0.7731	Fig. S8 (b) vs (d)
	Outdoor	119	-0.19	30.44		
Growth CO_2	Indoor	174	-0.01	35.97	0.7753	Fig. S9 (b) vs (d)
	Outdoor	119	0	26.09		
T_{leaf}	Indoor	174	1.31	-3.28	0.6165	Fig. S10 (b) vs (d)
	Outdoor	119	1.54	-15.55		
PPFD	Indoor	174	0.01	14.61	0.0623	Fig. S11 (b) vs (d)
	Outdoor	119	0.01	13.03		

Table S3. Results of multivariate linear mixed-effects models testing the response of V_{pmaxA} and A_{max} against species-specific traits and experimental conditions for **data with *PPFD* > 1000 μmol photons m^{-2} s^{-1}**. *P* values of fixed effects and their interactions are shown. See Table 1 and main text for more details. Effect size denotes the number of studies, while the sample size is the number of V_{pmaxA} or A_{max} data points. Continuous variables (i.e. mean T_{max}, growth CO_2, T_{leaf} and *PPFD*) were mean-centred before the analysis. Model's marginal R^2 reflects the variance explained by fixed factors only, whereas conditional R^2 considers the variance explained by both fixed and random factors. Bold text indicates statistical significance. Significant codes: *, $P < 0.05$; **, $P < 0.01$; ***, $P < 0.001$.

	V_{pmaxA}	A_{max}
Fixed effects (*P* values)		
C_4 subtype	0.764	0.864
Growth form	0.294	0.760
Growth location	**0.013***	**0.030***
Mean T_{max}	**0.002****	**0.003****
Growth CO_2	0.097	0.542
T_{leaf}	**< 0.0001*****	**< 0.0001*****
PPFD	0.119	0.061
C_4 subtype × T_{leaf}	0.795	0.702
C_4 subtype × Mean T_{max}	0.960	0.744
C_4 subtype × *PPFD*	0.704	0.795
Growth form × T_{leaf}	0.219	0.263
Growth form × Mean T_{max}	0.625	0.692
Growth form × *PPFD*	0.097	0.521
T_{leaf} × Mean T_{max}	0.278	**0.002****
Species group	0.179	0.162
Overall model statistics		
Effect size/sample size	43/317	34/261
Marginal R^2	0.50	0.41
Conditional R^2	0.80	0.72
